# Surface and bulk mechanisms in repeating treatment of solid surfaces by purified water

**DOI:** 10.1016/j.heliyon.2023.e17163

**Published:** 2023-06-09

**Authors:** Andriani Tsompou, Vitaly Kocherbitov

**Affiliations:** a Department of Biomedical Science, Malmö University, Malmö, Sweden; b Biofilms Research Center for Biointerfaces, Malmö University, Malmö, Sweden

**Keywords:** Washing and cleaning, Water purity, Quartz crystal microbalance with dissipation monitoring, Temperature, Washing cycles, Mechanisms of washing and cleaning, Emulsification, Olive oil, Surface tension, Entropy, Charge stabilization

## Abstract

To decrease the negative impact of surfactants, the idea of using purified water in washing has been proposed. Previous studies showed that purified water facilitates the roll-up mechanism by promoting electrostatic interactions between the surface and the soil. However, washing mechanisms can be dependent on the amount of remaining soil.

In this work we studied the removal of thin Vaseline films and thicker oil films from hydrophilic surfaces using multiple washing cycles at different temperatures. The Quartz Crystal Microbalance with Dissipation monitoring (QCM-D) and gravimetric analysis were used for thin and thick films respectively. In QCM-D experiments most of the thin film was removed during the first two cycles, while following cycles did not substantially affect washing efficiency; increased temperature facilitated the washing process. Gravimetric analysis showed that the washing of thicker films can be divided into two regimes. During the first, exponential, regime the amount of oil on the surface is high and surface mechanisms, such as roll-up, dominate. Oil droplets are kinetically stabilized in purified water by electrostatic interactions. As the amount of oil on the surface decreases, the second, linear, regime is introduced. The removal of oil occurs by equilibrium bulk mechanisms, where electrostatic interactions are less important.

## Graphical abstract

**Figure:**
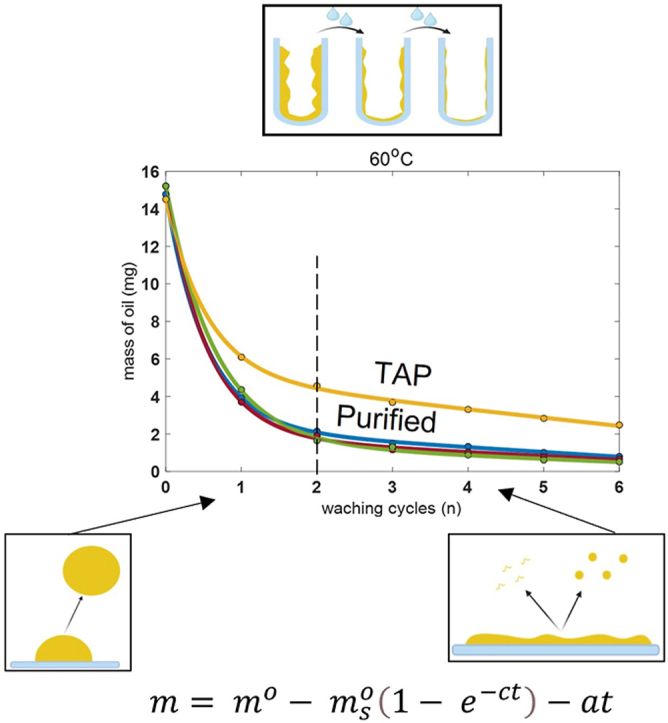

## 1 Introduction

Detergency is the process where soil is removed from a substrate material [1]. For more than 100 years, synthetic surfactants have been employed as the primary active agents in this process [2], and they are considered suitable for many domestic and industrial applications, such as washing and cleaning [3]. Nowadays, additives such as builders (*i.e*., zeolites, phosphates), are used to optimize the process. The environmental consequences of the combination of these chemicals in detergent formulations remain partly unknown [2]. Addition of phosphates can cause major water pollution and phosphates are known to affect the aquatic life of the recipient [2,[4], [5], [6]], while the effect of many biodegradable products on the environment is still unknown [7]. Also, their effect on human health has not been unnoticed [[8], [9], [10], [11], [12], [13], [14], [15], [16]].

The constant increase of reports informing about the ever-increasing risks of detergents have led scientists to find more environmentally friendly washing techniques. A great part of this research is focused on developing more eco-friendly surfactants based on natural sources [3,6,17,18]. De-gassed water [[19], [20], [21]] and addition of nanoparticles [22] are alternative experimental methodologies.

Another, bolder approach, that started coming up the past years, is the complete elimination (or major decrease) of surfactants and the use of ultrapure water for washing and cleaning [23,24]. The effectiveness of ultrapure water has been detected in various technical reports [14,23] but without discussion of the mechanism behind the washing effect. To understand better this washing approach, the actual washing mechanism should be understood.

Understanding the mechanism for cleaning without detergents is a difficult task as many factors should be considered *i.e*., surface and soil properties, water properties, washing time, and temperature of washing/cleaning *etc*. [[25], [26], [27]]. Soils can be defined as unwanted substances that make the surface unclean [27]. They can be water soluble or insoluble. Water soluble soils can be easily removed from a surface by water, whereas water insoluble liquid soils, better known as oily soils, consists of hydrocarbons, fatty acids (saturated and unsaturated), esters of fatty acids and alcohols [27] and their removal can be a challenge. Their properties, such as the viscosity [28,29], polarity, and solubility are important for detergency. Some of these properties are dependent on the water temperature, and when the temperature changes, these properties are also altered, making it easier or harder for the soil to be removed; *e.g*., higher temperatures can reduce the viscosity, making it easier to remove the dirt [30].

Although the removal of soils is highly relevant for cotton or synthetic fabrics, most of the studies regarding detergency have focused on the removal of substances from flat surfaces with the assumption that the mechanisms are identical [30]. In practical situations, oily soils are trapped inside the fibrous mesh, making it harder to devise accurate and reproducible experimental setups.

The mechanisms governing detergency have been discussed and described in the literature [26,[31], [32], [33]]. The main problem to be solved is the interactions between the surface and the soil. The work needed to remove the soil from the surface is called the work of adhesion and is related to the interfacial tension of soil/surface, soil/medium, surface/medium [32]. In the roll up mechanism, the driving force that causes the soil removal is an imbalance of the interfacial tensions at the three-phase contact line [26,32] Through Young’s equation, the interfacial tensions are related to the contact angle of the oil droplet [30,32,34]. In the ideal case, when the contact angle is 180°, the droplet will be spontaneously removed. If the contact angle is lower than that, mechanical energy is required. In the case where a small amount of oil remains on the surface, an effect called ‘necking’ can occur. In this case, the soil will be partially detached from the surface [26,35]. Another important mechanism is the emulsification process in which mechanical energy helps to emulsify a part of the soil. Finally, the solubilization mechanism describes the ability of oil molecules to be dissolved in bulk water or in surfactant micelles [27,36,37].

When detergents are present, surfactants will facilitate all three mechanisms. Accumulation of surfactant on the surface will decrease the soil/medium and surface/medium interfacial tensions thus the roll-up mechanism will be facilitated. Formation of kinetically stable emulsions can decrease the re-deposition of soil on the surface; hence the emulsification mechanism is facilitated. Formation of surfactant micelles helps in solubilizing insoluble soils by diffusing them into the hydrophobic cores of micelles [26,32,36].

The existence of more than one removal mechanism prevents establishing of a simple relationship between soil removal and contact angle or interfacial tension, which are important factors for the mechanisms. Thompson [25] examined water/oil/surfactant systems and showed that there is a linear relationship between the percentage of oil removed and the contact angle of the given oil. However, when the surfactant was changed, the dominant oil removal mechanism changed and the linear relationship also changed. Dillan et al. [38] also linked the interfacial tension to the roll-up removal mechanism. An important problem still exists: each system needs to be examined separately since a general relationship between the mechanisms is yet to be established [30].

The rate and the amount of solubilization are important when oily soils are removed from a surface [39]. Many studies have focused on these aspects when different surfactants (anionic, nonionic and mixtures of them) are used for cleaning [1,26,30,31,38,[40], [41], [42], [43]]. Hence, the rate of solubilization will increase with increasing surfactant concentration and temperature. Whereas the rate of solubilization decreases when the molecular weight of the hydrocarbon or the oil increases [40]. Some of these studies showed that the maximum solubilization was found when the soil was in an intermediate phase (microemulsions/liquid crystalline phase) which can develop during the washing process [30].

In a previous study, when the cleaning process was investigated without the use of surfactants [23], we suggested that ultrapure water facilitates the roll-up mechanism, by increasing the electrostatic interactions between a hydrophobic substance and a hydrophilic surface. The absence of ions in purified water increases the electrostatic repulsion between soil and surface and facilitates soil removal [23]. Moreover, the contact angle increased in the absence of ions. With the addition of mechanical energy, the oil was completely removed from the surface.

When Vaseline was used as a hydrophobic substance, QCM-D experiments showed that purified water exhibited more than 90% efficiency when one washing cycle (40 min) was used at 25 °C [23]. Although the value is high, complete removal of soil is needed. For that, the mechanisms of washing and cleaning needs to be examined further. A factor that affects the solubility is temperature, thus, to explore the mechanism behind washing and cleaning with ultrapure water, the temperature of the washing process is going to be examined. Another important factor for detergency is the washing time. Studies have shown that different detergents are able to clean a surface in different times [38,42], implying that throughout the washing process the kinetics continuously change.

Evaluation of detergency can be achieved through many different methods such as colorimetry, spectroscopy, fluorescence and/or assessment by the human eye. Another approach that has been used before is the Quartz Crystal Microbalance with Dissipation method which can monitor real time changes occurring on the surface under examination [23,44].

In this work, we present and compare the effect that temperature and the number of washing cycles have on the washing efficiency when purified and non-purified water grades are used in cleaning hydrophilic surfaces. We use QCM-D experiments to study the washing efficiency of thin hydrophobic films and gravimetric analysis to study the efficiency of removal of thicker films. Based on these experimental results, we try to better understand the mechanism behind washing with purified water grades and how the detergency mechanisms work together in this system.

## 2 Materials and methods

### 2.1 Materials

For the following experiments, four different water grades and one solution were used, and they will be referred to as follows:

• MQ: Milli-Q Water purified using a Millipore Milli-Q lab water system. It is produced in the laboratory using PURELAB flex (ELGA, UK)
• DIRO: ultra-pure water provided and produced by SWATAB (Malmö, Sweden)
• ΤΑP: tap water from the university building, Malmö, Sweden
• NaCl: 10 mM NaCl solution in MQ water. Concentration will be specified if different than 10 mM
• SDS: 4 g/L Sodium dodecyl sulfate solution. The substance was purchased from Sigma-Aldrich, Germany

Two different types of hydrophobic substances/materials were used:

• Olive oil: Extra virgin olive oil (FONTANA est 1978, classic, Spain)
• Vaseline (density 0,94 g/cm³, kinematic viscosity 7,5–10 mm^2^/s at 100 °C) purchased form Carl ROTH

MQ water was directly used for the experiment as the purification system is available in the laboratories. DIRO water was stored in 20 L plastic bottles while the NaCl solutions were stored in glass bottles.

### 2.2 QCM-D method and film deposition

QCM-D method has been around since the 1960s for monitoring with high sensitivity the mass and the thickness of a material adsorbed on a sensor surface. The core of this technology lies on the piezoelectric quartz sensors *i.e*., a material where an applied electrical field will give rise to a mechanical deformation. In practice, when voltage is applied, the sensor will oscillate back and forth in synch with the voltage that is applied. This oscillation will create an acoustic wave, where the resonance frequency can reveal the thickness of the (un)coated sensor.

All experiments were performed using the Q-Sense QCM-D E4 unit equipped with a standard flow module (Biolin Scientific AB, Sweden). The sensors used for the experiments are the QSX 303 SiO_2_ QCM-D (5 MHz) sensors from Biolin Scientific AB (Sweden). All sensors were cleaned in accordance with the manufacturer’s recommendations.

The films were deposited on the sensor by spin coating. To coat the sensors the following procedure was used: Vaseline was diluted 110 times in toluene. 14 μl of the sample were deposited on the sensor surface while the sensor was rotated in 1200 rpm (the spin-coater was developed in house). The sensor was left to dry in the fume hood for 15 min and another 30 min in a desiccator.

A ±20 Hz error can be produced by mounting and remounting of the sensor in the QCM-D module. To reduce this inaccuracy all measurements in air were performed five times. The washing efficiency was calculated by measuring the thickness of the film before and after each washing cycle.

For calculations based on the Sauerbrey equations (see below), the mean frequency values with their standard deviations were used.

With the QCMD method, two important parameters for washing were examined. These are the water temperature and the number of the washing cycles. All further experiments were done with 4 washing cycles. Each cycle consists of washing the sensor for 15 min and measurement of the thickness after washing. The temperatures examined were 25 and 40 °C in both the water and the QCMD unit. The water temperature was regulated by a water bath throughout the experiment. The pump speed was 0.25 ml/min. Experiments were also conducted with 0.5 ml/min (supplementary data, Fig. S10) but due to inconclusive results, all discussed data was acquired with a pump speed of 0.25 ml/min.

The QCM-D experiment was initiated according to a procedure described in detail elsewhere and slightly changed to fit the current experimental questions [23]. In brief, the bare sensor was measured in air 5 times and after that it was spin-coated as described above. The thickness of the layer was measured in air 5 times. Next, the first washing cycle began, *i.e*., one water grade (MQ, DIRO, TAP, NaCl) was passed through the unit at the given temperature (25 or 40 °C) for 15 min. The sensor was then allowed to dry, and the thickness of the layer would again be measured 5 times in air. The same process was repeated three more times. After each washing cycle, an optical microscope was used to visualize the removal of Vaseline from the surface. Images were taken with a 10× lens. The scalebar in the images is 100 μm. A minimum of 3 images were collected from each sample.

### 2.3 QCM-D data analysis

The obtained data was analyzed as described in our previous work [23]. The Sauerbrey equation [45] was used to calculate the mass of the dry film:

(1) $$\Delta f_{n}=-\frac{2{f_{0}}^{2}m}{Z_{q}}$$

and thickness was calculated as:

(2) $$th=\frac{V}{A}=\frac{m}{Ap}$$

were $\Delta f_{n}$ is the frequency change normalized per odd overtone number *n*, *m* is the areal mass (kg m^−2^), *Z*_*q*_ is the acoustic impedance of quartz (*Z*_*q*_ = 8.8 10^6^ kg m^−2^ s^−1^), *f*_*o*_ is the fundamental resonance frequency of the quartz sensor (≈5 MHz), *t* is the thickness of the dry film, *V* is the volume, *A* is the area, *m/A* is the Sauerbrey mass and *ρ* is the density. A density of 940 kg/m^3^ was used for Vaseline. The standard deviation for a frequency change was calculated as follows:

(3) $$std\left(\Delta {\bar{f}}_{n}\right)=\sqrt{{\left[std\left({\bar{f}}_{n}c\right)\right]}^{2}+{\left[std\left({\bar{f}}_{n}b\right)\right]}^{2}}$$

where ${\bar{f}}_{n}c$ and ${\bar{f}}_{n}b$ are mean frequencies for coated and bare sensors respectively, averaged over a certain time.

Viscoelastic modeling of films in liquids were performed using QTools.

### 2.4 Gravimetric analysis: measurement of oil film mass before and after water contact (washing cycles and temperature effect)

To detect the effect that different temperatures and multiple washing cycles have on a glass surface, the oil film mass before and after contact with water was measured. The experiment was performed on 15 mL glass tubes and after each step the mass of the tube was measured with a Mettler Toledo AT261 analytical scale. First, 14–15 mg of olive oil was placed on the tube surface and with the help of a cotton stick the oil was spread on the whole surface. 5 g of water were then added. The tubes were vortexed for 10 s and the excess water was discarded. The tubes were left to dry overnight in the freeze dryer ALPHA 1–4 LCS (temperature: −60 °C, pressure: 0.08 mbar) and their mass was measured the following day.

The temperatures of the water grades that were examined were 25, 40, and 60 °C and were controlled via a thermostat (LAUDA, ECO Gold, Germany). The number of washing cycles performed was between 1 and 11. At each temperature, a maximum of 11 washing cycles was performed for each water grade.

### 2.4 Ion concentration and conductivity measurement in different water grades

To determine the sodium and calcium concentration in the different water grades, two ion selective electrodes (8220BNWP and 9720BNWP from Thermo Scientific) were used on the Thermo Scientific Orion Star A214 Benchtop pH/ISE meter (Gothenburg, Sweden). A calibration of the electrode was done by using five NaCl solutions with concentrations 2, 1, 0.5, 0.25 and 0.125 M. Calibration curves were made as recommended by each manual and between each solution the electrode was rinsed with distilled water. The concentration of the ions was then measured for all water grades in molarity (M), following instructions from the manual. For the conductivity measurements the Bench conductivity/TDS Meter CON 510 (Eutectic instruments, Singapore) was used. The measurement method is described in detail in our previous work [23]. Six replicates were performed for each water grade for every electrode.

## 3 Results

### 3.1 Characterization of water grades

Purified and non-purified water grades differ mainly in the concentration of ions and the impurities that are found in them. Since the main question in our research deals with the importance of purified water grades and their ability to wash without detergents, knowing their differences is important. Conductivity is an easily measured indicator of ion presence while the amount of sodium and calcium are the two ions that can categorize the type of TAP water that is being used through its hardness [46]. Purified water grades should not include any traces of neither of the ions in question here. Table 1 shows the conductivity and ion presence in all water grades. Conductivity values agree with the values found in previous work [23]. Conductivity values are higher than the ones that are found in the Malmö region. Sodium concentration is found to be lower than the corresponding value in the supplier's data [47]. Calcium concentration though is higher than the literature value [47].

**Table 1:** Conductivity (μS/cm), sodium and calcium concentrations in the liquids.

	Conductivity (μS/cm)	Sodium concentration (mM)	Calcium concentration (mM)	pH
MQ	0.19 (±0.04)	0 (±0)	0 (±0)	7.01
DIRO	1.01 (±0.08)	0 (±0)	0 (±0)	7.33
TAP (this work)	352.8 (±4)	0.8 (±0.1)	3 (±0)	8.02
TAP (supplier’s data)	107–140	1.6	0.9	8.2
10 mM NaCl	1029 (±14)	9.99 (±3.02e^−5^)	0	5.10

The difference in the conductivity values between MQ and DIRO depends on the purification system and storage. MQ water is measured directly from the purification system while DIRO water is being stored in plastic 10 L bottles for longer periods of time.

### 3.2 Quartz Crystal Microbalance with Dissipation (QCM-D)

#### 3.2.1 Monitoring removal of hydrophobic films in different temperatures by water using QCM-D

**Fig. 1:**
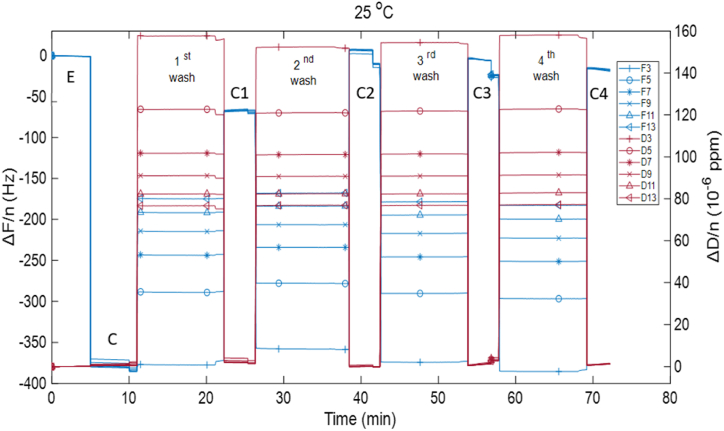
Frequency (blue) and dissipation (red) shifts for overtones 3,5,7,9,11,13 obtained from QCMD-D measurement of MQ water at 25 °C for 4 washing cycles. The regimes are: E – empty (bare) sensor in air, C – sensor coated with a Vaseline thin film in air, C1:C4 – dried sensor in air after treatment with water, 1st:4th wash – coated sensor in MQ water. (For interpretation of the references to color in this figure legend, the reader is referred to the Web version of this article.)

The QCM-D method was used to investigate the removal of thin Vaseline film from the silica surface. The raw data of the experiments conducted with MQ water are presented in Fig. 1. To assess the importance of temperature in washing and cleaning, two different temperatures were used. Both the QCMD machine and the water used were constantly either at 25 or 40 °C. Due to machine limitations, higher temperatures could not be measured. The experiment consists of 4 main regimes. 1 – bare sensor in air, 2 – sensor coated with Vaseline film in air, 3 – sensor with Vaseline film in water, 4 – sensor after treatment with water and drying. Regimes 3 and 4 are the actual washing cycles, and thus both of them occur 4 times (*i.e.*, the number of washing cycles for each experiment). As mentioned in our previous study [23], when the medium changes from air to liquid and vice versa a stepwise frequency shift occurs. Moreover, in both temperatures, the film thickness after the washing cycles is substantially lower than the coated, non-washed layer.

Comparing the raw data for the two different temperatures, it can be observed that during the washing step, where the surface is in contact with the water, both frequency and dissipation show an increasing and decreasing trend, respectively. Regarding the experiments at 25 °C, as mentioned in previous work [23], although properties of the washing step is difficult to analyze using the raw data, it is known that the important part in the washing process is when the interface changes from solid-air to solid-liquid. In this work we confirmed this finding using QCM-D data modeling which allows assessment of film thickness in both liquid and air (see Supplementary data).

#### 3.2.2 Behavior in air and washing efficiency

**Fig. 2:**
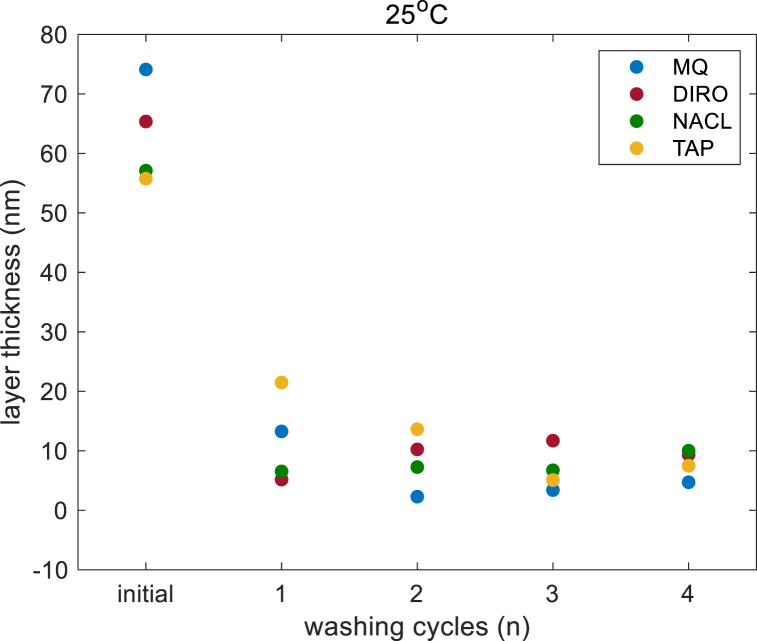
Film thickness after each washing cycle at 25 °C, calculated with the Sauerbrey equation.

To find the amount of Vaseline that was removed after each washing cycle and to detect efficiencies at different temperatures, we calculated the film thickness using Sauerbrey equation for rigid film (eqs (1) and (2)). Since all requirements for a rigid film were met (supplementary data), any calculation with the viscoelastic model could induce an error in calculations. All measurements in air were done five times to provide better statistics (eq (3)) and decrease the effect of re-mounting the sensor (see Methods section). Fig. 2 shows the calculated thickness after each washing cycle at 25 °C when the Sauerbrey equation was used while Table S2 shows the calculation of the film thickness when the viscoelastic model was used. The difference in the values is negligible.

**Fig. 3:**
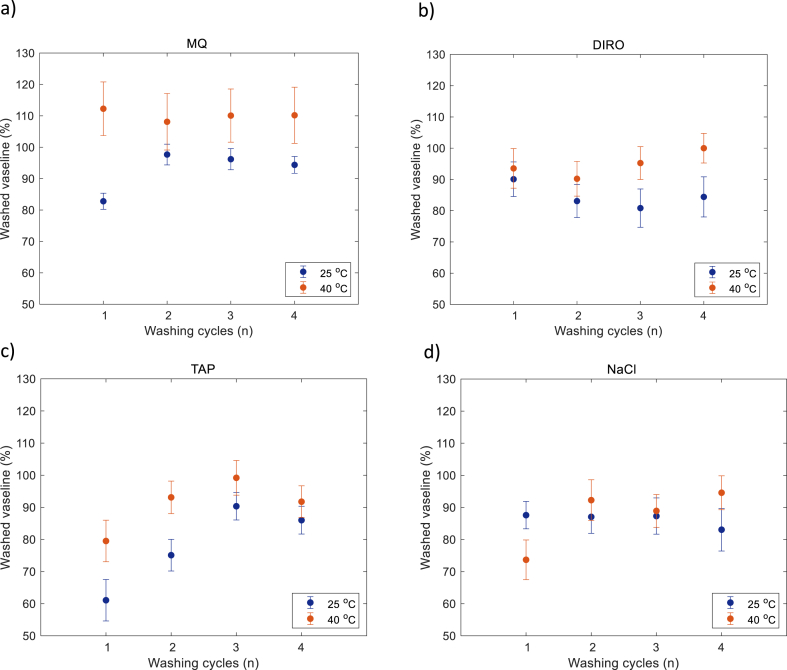
The cleaning efficiency: fraction of washed off Vaseline on QCM-D experiments when different water grades a) MQ b) DIRO c) TAP d) NaCl were used at 25 °C (blue) and at 40 °C (red). (For interpretation of the references to color in this figure legend, the reader is referred to the Web version of this article.)

For each temperature, the surface was exposed to 4 washing cycles (10 min each). Fig. 3 shows the efficiency (%) for each water grade at each temperature. Regardless of the water grades, the efficiency is higher when the temperature increases. In all four water grades 100% efficiency is reached, but after a different number of cycles.

For each water grade examined, the efficiency is higher with higher temperature. In MQ water at 25 °C, maximum efficiency is reached after the second washing cycle, while at 40 °C, the surface seems to be clean after the first cycle. In DIRO water, the results follow the same trend during the first 2 cycles. At increased temperature, the efficiency is increased at the last two cycles. When NaCl was used, the efficiency after the first washing cycle seems lower at higher temperature but quickly increases after the second cycle. At 25 °C, the efficiency did not reach the maximum even after 4 cycles. At 40 °C, the surface seems to be clean after the last washing cycle. In TAP water a gradual increase in the efficiency is seen for both temperatures. When 25 °C water was used, the sensor was not completely clean at the end, while the surface appears to be clean after 3 washing cycles at 40 °C.

To visually investigate how the surface looked after each washing cycle, photos were obtained after each step (Fig. S12). Residues are visible after the 4th cycle when non-purified water grades were used. Moreover, the increased washing efficiency at higher temperature was evident from the photos.

### 3.3 Gravimetric experiments with glass tubes

From QCM-D experiments with thin Vaseline films (less than 80 nm) we observed that after only one washing cycle almost all Vaseline is washed away. For thicker films (corresponding to the mass of 15 mg), a single treatment was insufficient. Therefore, further developing the approach presented in our previous work [23], we investigated the effect of the number of washing cycles, water purity, and temperature on cleaning of a thick olive oil layer from a hydrophilic surface. Since glass can withstand higher temperatures than the QCM-D unit, an additional temperature, 60 °C, was included. The number of washing cycles was increased to 6.

#### 3.3.1 Effect of water purity

**Fig. 4:**
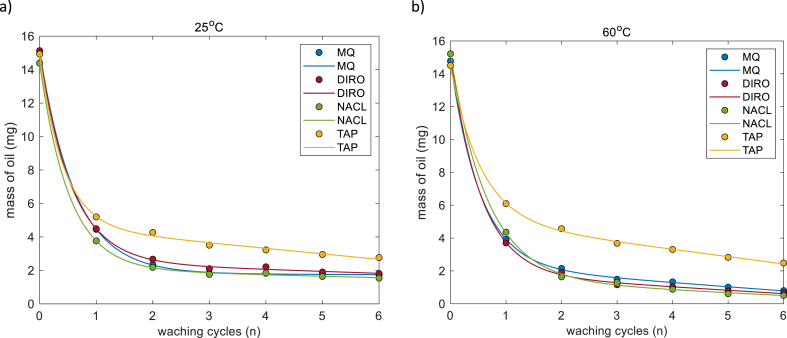
Average amount of olive oil left on the surface of glass tubes (mg) as a function of the number of washing cycles for MQ, DIRO, NaCl, and TAP at a) 25 and b) 60 °C. The number of replicates is 3. Plots with error bars can be found in the supplementary data.

Fig. 4 shows the results of gravimetric experiments (the amount of oil remaining after a washing cycle). All water grades show qualitatively similar behavior: after the first cycle more than half of the oil is removed, further cleaning cycles result in a gradual decrease of the remaining amount of oil. Moreover, MQ, DIRO, and NaCl samples show quantitatively similar behavior and the difference between these samples is smaller than the error bars (see Supporting info).

In contrast, TAP water exhibits a quantitatively different behavior in comparison to the other three grades. Fig. 4 and Fig. S14 shows that at all temperatures, treatment with TAP water leaves the highest amount of oil on the surface, hence its efficiency is lowest (this effect is particularly clear for 60 °C).

#### 3.3.2 Effect of number of washing cycles

The effect of the number of washing cycles can be found in Fig. 4. In all cases, there is a decrease in the mass with respect to the washing cycle number *n* regardless of the temperature or the water grade. The overall behavior can be divided into two regimes: the first regime includes the first two washing cycles (points 0–2 in the plots) where a strong non-linear decrease of the amount of oil is observed. In contrast, during washing cycles 3–6, a linear decrease of mass is observed, and the removal of oil is slow.

To better understand the difference between the two washing regimes, the data was non-linearly fitted with a dependence that includes an exponential term (dominating the initial steps of the process) and a linear term (dominating steps 3–6). The equation describing the time dependence is:

(4) $$m=m^{o}-m_{s}^{o}\left(1-e^{-ct}\right)-at$$

were m is the mass of removed oil (mg) after the washing cycle, $m^{o}$ is the initial mass of oil (mg), $m_{s}^{o}$ is the amount that can be removed during the exponential regime (mg), c is the exponential decay constant, a is the slope of the linear dependence, and t is the washing time in a continuous washing process. Since in the experiments we used a procedure where we interrupted the washing process to measure the mass of remaining oil, it is more convenient to re-write this equation in terms on the number of the washing cycle n:

(5) $$m=m^{o}-m_{s}^{o}\left(1-e^{-cn}\right)-an$$

Both equations combine the exponential and the linear part of the washing process. The exponential part, $m_{s}^{o}\left(1-e^{-cn}\right)$ , describes the fast oil removal in the initial steps while the linear part, an*,* describes the slow oil removal that dominates the final steps of the washing process. Although a detailed discussion of mechanisms involved in removal of oil will be presented in the Discussion section, here we will try to make a preliminary assignment of the exponential and linear terms in the equations above to possible washing mechanisms.

When water is added to the system during the washing process, part of the oil will be solubilized or dispersed into the medium. Although, the removal of oil via this mechanism is slow, it occurs throughout the whole washing process. According to equilibrium thermodynamics, in a two-phase situation, the amount of oil that can be dissolved in the aqueous phase is not dependent on its amount in the oil phase. Hence, addition of the same amount of water in each washing cycle should result in removal of constant amount of oil, in other words a linear dependence of mass vs number of washing cycles. It can be linked to the slope a through eq (6). The slope equals to the mass difference between n+i and n washing cycles, as indicated:

(6) $$a=\frac{m_{n+1}-m_{n}}{\left(n+1\right)-n}=\Delta m$$

From this slope, the apparent bulk solubility, $C^{app}$ in mg/g (which for current conditions is close to g/L), can then be calculated:

(7) $$C^{app}=\frac{a}{m_{t}}$$

here $m_{t}=5.015\;g$ is the total mass of the system.

**Fig. 5:**
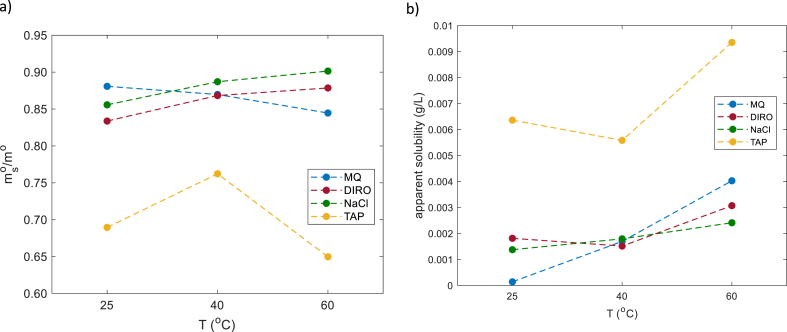
Parameters calculated from the non-linear fit of gravimetric data obtained from glass tubes for MQ, DIRO, NaCl and TAP at 25, 40 and 60 °C. a) mass of oil removed during the exponential regime b) apparent solubilities (g/L) obtained using equation (7).

The remaining part of eq (5) (exponential part) is not related to the bulk solubility but to a faster oil removal mechanism involving the surface. The dominant surface mechanism is the roll up mechanism and it is known that the roll up mechanism can remove a large amount of oil from the surface. Dividing $m_{s}^{o}$ by $m^{o}$, one can calculate the fraction of oil that was removed through the surface mechanism. With a maximum value of 1 (when everything is removed), the contribution of the roll up mechanism (calculated from the non-linear fit) is shown in Fig. 5a.

Fig. 5a indicates that when MQ, DIRO and NaCl grades are used, the roll up mechanism contributes to a high degree. These samples show quantitatively similar results and the difference amongst them is small. TAP water shows different quantitative results.

Regarding the exponential decay and the slope, all water grades exhibit qualitatively the same behavior (Figs. S16 and S17). TAP water exhibits a quantitatively different behavior in comparison to the other three grades. To determine the effect that solubility has in this system, its value was calculated and shown in Fig. 5b. Since olive oil has a low solubility in water and it is hard to determine its exact value, the solubility found here will be called apparent solubility. Regarding the apparent solubility values, TAP water exhibits the highest values. In all cases, a temperature dependent is found. As the water temperature increases, the apparent solubility is increased.

#### 3.3.3 Effect of temperature

**Fig. 6:**
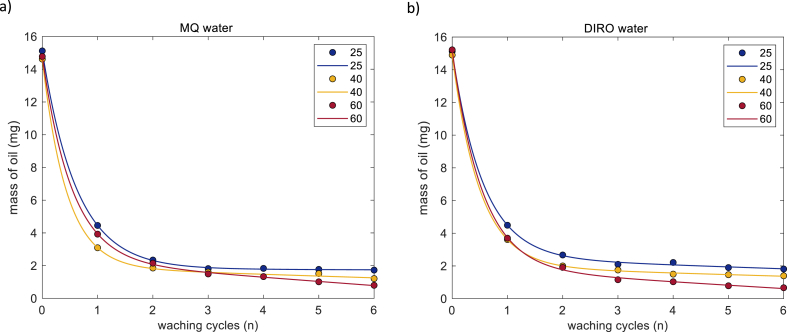
Average amount (mg) of oil left on the surface after each washing cycle when different water grades (a) MQ (b) DIRO were used at 25 °C, 40 °C, and at 60 °C. Data is non-linearly fitted using eq (4). The number of replicates is 3.

The effect of temperature was studied by plotting the results of each water grade at different temperatures (Fig. 6, Fig. S18). MQ, DIRO and NaCl samples show quantitatively similar behavior at all temperatures. Fig. 5a, shows that the amount of oil that is removed from the surface via the surface mechanism increases at each temperature.

An effect of the temperature is also present during the linear regime. As the temperature increases, the amount of oil that is left on the surface is decreasing (Fig. 6, Fig. S18). Fig. 5a shows that the apparent solubility also increases with increased temperature. In all three cases, the highest efficiency of the linear regime was found at 60 °C.

TAP water, has qualitative the same behavior as the other grades, but in both regimes the highest efficiency was found at 40 °C instead of 60 °C. At 25 and 60 °C, the behavior of TAP water is similar with a small difference at the removal rate (supplementary data). Fig. 5 points out the different behavior of TAP water.

## 4 Discussion

### 4.1 Thin film removal

In our previous study we showed that water can remove hydrophobic soil from a surface by penetrating between the oil and the surface. This creates new interfaces that decrease the Gibbs energy of the system. Moreover, we showed that disjoining pressure is of importance and its increase can be correlated to the lowering of the salt content [23].

In the current QCM-D experiments, at both temperatures, purified water grades were able to remove the soil from the surface faster than NaCl and TAP water. This data is in agreement with the QCM-D data from our previous work [23] and it shows that thin hydrophobic layers can be easily removed from a hydrophilic surface by treatment with purified water as it enhances the disjoining pressure and provides sufficient repulsion between the negative charged oil-water interface and the negative charged silica surface [23]. The presence of ions either from NaCl or TAP, decreases the repulsion.

Although the efficiency of non-purified water grades reached high values, this occurred only when a higher number of cycles was employed. To ensure that the surface was getting washed progressively after each cycle, photos of the surface were obtained (Figs. S12 and S13). After the 4th cycle, residues were still visible in the experiments with NaCl and TAP and they could either be small amounts of Vaseline or precipitated salts.

When higher temperatures were used, the efficiency of all water grades increased. These results are easily explained from a point of view of the soil properties. It is known that when the temperature increases, the viscosity decreases, making it easier for the soil to be removed [27]. In QCM-D, Vaseline was used as the hydrophobic substance and its melting point varies between 38 and 60 °C since its composition may vary. The Vaseline that was used in these experiments has a melting temperature of 50 °C [48]. This suggests that when the surface is being washed at 40 °C instead of 25 °C, the fluidity of the Vaseline should to some extent increase, which could make the removal easier.

When SDS solutions of different concentrations were used to clean the surface, the efficiency was again high, as previous work indicated. Interestingly, after only one washing cycle, small crystalline structures were observed at the surface, indicating precipitation of SDS, even when the concentration used was below the critical micelle concentration.

### 4.2 Effects of water purity in removal of oil from solid surfaces

The data presented in section 3.3 shows that thick oil films can be removed from a solid hydrophilic surface and the efficiency of the process is affected by the salt content. The effect of salt can be described by the DLVO theory (Derjaguin-Landau-Verwey-Overbeek theory) that defines the stability of the system. DLVO theory describes the balance between repulsive and attractive forces that act on the system. Two types of forces are considered. A long-range attractive van der Waals force that acts irrespectively of the chemical properties of the medium, and a repulsive electrostatic double layer force that is dependent on the ionic strength of the medium and polar features of interacting entities [31,49,50]. These forces are independent of one another and together they control the stability of charged surfaces [51].

A system is considered stable when a high enough energy barrier is created that does not allow the particles to coagulate [52]. The simplest mean of controlling the stability and the energy barrier is by changing the concentration of the ions found in the system. This can be done by altering the salt content, affecting only the electrostatic forces. In our previous work [23], we demonstrated that ultra-low salt content increases the energy barrier and promotes stability.

To better understand the origin of the electrostatic forces in our system, the properties of the soil and the surface will be discussed briefly. Olive oil consists of triglycerides (99%), free fatty acids, mono - and diacylglycerols, and an array of substances such as hydrocarbons, sterols, aliphatic alcohols, tocopherols, and pigments; of all olive oil components, free fatty acids can be negatively charged at neutral pH [53]. Regarding glass surfaces, they are known to acquire a negative surface charge when immersed in water, primarily through the dissociation of terminal silanol groups. The degree of dissociation and the density of surface charges is a result of the equilibrium that is formed between the counterions at the glass surface and the free ions in the bulk electrolyte [54].

DLVO theory can be used to describe interactions between dispersed spherical particles or flat surfaces and particles. In both cases, electrostatic interactions can promote stability when the salt content is decreased. In the first case, pure water can enhance the electrostatic interactions between the surface and the soil. The charge that exists on the surfaces in combination with the absence of ions in water leads to the detachment of the soil by increasing the soil to surface contact angle [23]. In case of a liquid soil, such as olive oil, a droplet can be formed on the surface and the contact angle will be altered depending on the purity of water. For the washing process, this phenomenon can be described by the roll up mechanism. Through this mechanism, purified water grades and NaCl with a low salt content can remove higher amounts of oil from the surface compared to TAP water. Apart from decreasing the electrostatic forces, divalent ions such as Mg^+2^ and Ca^+2^ present only in TAP water can form electrostatic “bridges” between free fatty acids and the solid surface, hindering the detachment process [55].

In the second case, with charged spherical particles in a medium, the purity of the water helps stabilize those particles in the system by increasing the Debye screening length. This charge stabilization prevents redeposition of soil on the surface.

The data presented here agree with our previous work on the effect of water purity. Additional information concluded from these experiments (Fig. 4, Fig. 5) is that pure water facilitates removal of oil during the first two washing cycles, while in the following cycles it is not clearly observed. To understand the reason for that, the features of the main washing mechanisms will be further discussed in the next part.

### 4.3 Surface and bulk mechanisms of washing

#### 4.3.1 Overview of washing mechanisms

**Fig. 7:**
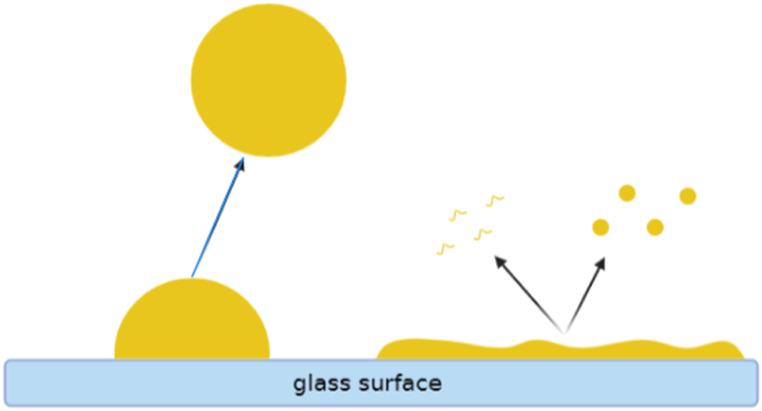
The mechanisms of oil removal from surface in water. Left: high amount of oil on the surface - big droplets can be removed when the oil contact angle is more than 90 °C (roll up mechanism). Right: lower amount of oil on the surface – solubilization of individual molecules or emulsification of oil can remove smaller amounts of oil.

As discussed in the introduction part, the main mechanisms that govern detergency are: 1) the so called roll up, 2) emulsification, and 3) solubilization. Fig. 7 shows how each mechanism can facilitate surface oil removal in water. As a surface mechanism, roll up depends on the interaction forces between the surface and the soil and can easily be described by changes in the contact angle. In solubilization, however, the tendency of the soil to be dissolved into the bulk is not dependent on the contact angle between the oil and the surface. Hence solubilization can be considered as a bulk mechanism. The properties of emulsification mechanism have certain similarities to the features of both roll-up and solubilization mechanisms.

The data obtained from the gravimetric analysis clearly pointed to the occurrence of two regimes, *i.e*., an exponential and a linear regime, and suggested an interdependence of the regimes and the background mechanisms. As mentioned in the results section, the exponential part can be correlated to the surface mechanism while the linear part is driven by the bulk mechanisms.

The roll-up mechanism, which was first described in 1937 by Adam [56], is the main surface mechanism and it can remove liquid droplets from the surface. The driving force causing the removal of the oil results from changes in the interfacial tensions between water, oil, and surface. The work that is required to remove a liquid droplet is called work of adhesion and is expressed as [32]:

(8) $$W_{ad}=\gamma_{ow}+\gamma_{sw}-\gamma_{so}$$

where $\gamma_{so}$ is the interfacial tension between surface and oil, $\gamma_{sw}$ the interfacial tension between surface and water, and $\gamma_{ow}$ is the interfacial tension between oil and water. At equilibrium the resulting interfacial tensions can be expressed by Young’s equation [32]:

(9) $$\gamma_{so}-\gamma_{sw}=\gamma_{ow}\;\cos\;\theta$$

where θ is the contact angle between the oil and the surface. By combining eqs. (8), (9)), the work that is required to remove the oil from the surface as a function of the contact angle of the oil droplets can be calculated:

(10) $$W_{ad}=\gamma_{ow}\left(1+\cos\;\theta \right)$$

For the oil droplet to be removed, the contact angle in eq (10) should be equal to 180°, or it can be between 90 and 180° if mechanical energy is used. The removal of films and oils from a surface can be discussed in terms of surface tension and contact angle or in terms of de-wetting and spreading coefficient [57]. In our previous work [23], we discussed the removal of thin Vaseline films in terms of surface tension, and the removal of oil in terms of contact angle. In case of oil, we concluded that the roll up mechanism is facilitated by the purified water grades by increasing the contact angle between the soil and the surface via enhanced electrostatic repulsions. Experiments with oil presented here are focused on kinetics in the situation when neither of the liquids fully wets the surface in expense of the other liquid and hence contact angle is the most appropriate parameter for characterization of the system. The data presented in this work support the earlier proposed theory, as in the exponential regime the efficiency of purified grades was higher.

Although the roll-up mechanism presents an effective way to remove the oil from the surface, it works best for removing large amounts of oil, while its efficiency in removing low amounts of liquid soil is limited [58]. When the volume of the droplet is low, the contact angle needs to be extremely high for the droplets to be removed, in contrast to the situation observed for high droplet volumes [26,58,59]. During the exponential regime, the volume of the oil is substantial to allow the formation droplets that relying on mechanical energy can partially or completely detach from the surface. Thus, big oil droplets can be removed via the roll up and necking mechanism and be kinetically stabilized in the solution by electrostatic interactions. In the experiments presented here, at the end of the exponential regime, the amount of oil left on the surface is greatly decreased and this results in the termination of the roll up mechanism.

The discussion presented above, shows that electrostatic interactions are important for the roll-up mechanism and for stabilization of large droplets in water. Below we will discuss if these interactions are equally important for smaller droplets and solubilization/emulsification mechanisms.

#### 4.3.2 Effect of the droplet size on electrostatic interactions

Gravimetric experiments indicate that during the linear regime, where the oil amount is low, the water purity is not the main factor that drives washing and cleaning. To understand how the system behaves/is stabilized when the presence of oil is low, the stability of the system as a function of droplet size needs to be considered.

As mentioned before, DLVO theory describes the stability of the system by combining the electrostatic repulsion forces and the attractive van der Waals forces. The electrostatic contribution $V_{R}$ to the total energy observed between two equal spherical particles is described as:

(11) $$V_{R}=\frac{32\pi \varepsilon ak^{2}T^{2}\gamma^{2}}{e^{2}z^{2}}\exp\left[-\kappa h_{d}\right]$$

where *z* is the ion charge, *a* is the particle size, ε is the permittivity of the dispersion medium, κ is the reciprocal Debye screening length, $h_{d}$ is the distance between the particles, and γ is defined by eq. (12) as

(12) $$\gamma =\frac{\exp\left[\frac{ze\psi_{d}}{2kT}\right]-1}{\exp\left[\frac{ze\psi_{d}}{2kT}\right]+1}$$

The London dispersion attraction energy $V_{A}$ for equal spheres can be written in terms of the ratio of distances $x=h_{d}/2a$ [60]:

(13) $$V_{A}=-\frac{A}{12}\;\left[\frac{1}{x\left(x+2\right)}+\frac{1}{{\left(x+1\right)}^{2}}+2\;\ln\left(\frac{x\left(x+2\right)}{{\left(x+1\right)}^{2}}\right)\right]$$

where *A* is the Hamaker constant. The sum of $V_{A}$ (eq (13)) and $V_{R}$ (eq (11)) defines the total energy of the system. The existence of an energy barrier provides information of the stability of the system. In our previous work [23], we demonstrated that the importance of charge stabilization greatly increases when pure water is used. The results on the exponential part of the cleaning process presented here confirm this theory.

**Fig. 8:**
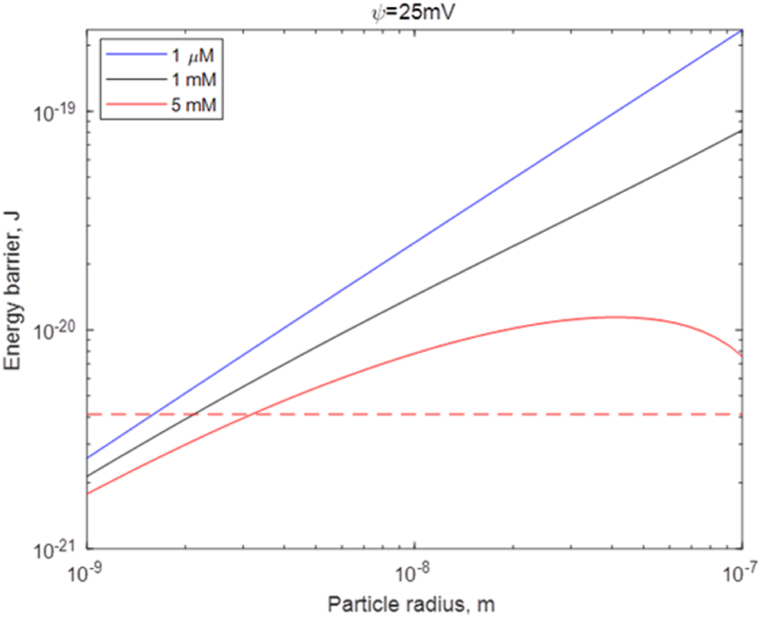
DLVO interaction energy barrier between spherical particles versus the particle size (m) at three different 1-1 electrolyte concentrations (1 μM, 1 mM, 5 mM) when the surface potential is stable at 25 mV. The dashed line corresponds to the thermal energy, *kT*. Other parameters are taken from D.J. Shaw, Introduction to colloid and surface chemistry, p 221 [60] and from D.F. Evans, and H. Wennerström, Colloidal Domain where physics, chemistry, biology, and technology meet, p 413 [52].

To further investigate the charge stabilization on different droplet sizes, we considered the DLVO energies for the case of different particle sizes. The results show that the energy barrier becomes smaller for smaller particles (this is summarized in Fig. 8 that shows the height of the energy barrier as a function of particle size, while Fig. S20 shows examples of energy-distance curves for a 1 μM 1-1 electrolyte). Moreover, regarding the smallest particles, the energy barrier becomes smaller than the thermal energy *kT* value, which demonstrates that they cannot be stabilized via electrostatic interactions. Noteworthy is the fact that for the smallest particles, a salt concentration variation by a factor of 5000 only slightly affects the energy barrier, in other words, the effect of salt on the stability of the smallest particles is very low.

A connection between the small droplets' sizes and the lower washing efficiency in the linear regime can then be made. By combining findings from section 4.3.1 and 4.3.2, we show that very small droplets cannot be stabilized via electrostatics, and hence other factors, associated with solubilization and emulsification mechanisms, play the main role in the linear regime.

#### 4.3.3 Dispersibility/solubilization of oil in water

In colloidal systems both thermodynamic and kinetic stabilization need to be considered in order to understand the state and evolution of the system. In our experiments, electrostatic interactions between the dispersed droplets increase the energy barrier and promote kinetic stabilization in the exponential regime (while the global energy minimum corresponds to coagulated system). For the linear regime, as we showed above, the energy barrier can disappear and the interactions between the oil and the water should be considered in terms of thermodynamic stability. For the system to be stabilized, the lowest energy state must be found. For that, the free Gibbs energy of the system as a function of the droplet size will be considered.

When oil is dispersed in water in a form of small droplets, the total Gibbs energy of this process can be written as follows:

(14) $$\Delta G=\Delta H-T\Delta S=\gamma_{ow}A_{ow}-T\Delta S$$

where $A_{ow}$ is the total oil-water interfacial area. Since the interfacial tension is positive, the only driving force for the dispersibility of oil can be an increase of entropy compared to the situation when oil and water are fully macroscopically separated.

Intuitively, one should expect an increase of entropy when the oil phase is dispersed in small droplets that can be distributed in a large volume of water. Finding a quantitative relation for the entropy change of formation of droplets is, however, a non-trivial task. One approach is to consider water as an unstructured medium and use expressions for the excess entropy of hard spheres, based on the Carnahan-Starling [61] equation:

(15) $$\frac{S^{ex}}{kN}=-\frac{4\varphi -3\varphi^{2}}{{\left(1-\varphi \right)}^{2}}$$

where φ is the volume fraction of spheres. The total entropy S of the system should then consist of an ideal part, $S^{id}$ and the excess of entropy, $S^{ex}$. Since we consider formation of droplets rather than their dilution, the equation for the ideal entropy should not be based on ideal mixing equations, but rather on the entropy of an ideal gas, *i.e*., the Sackur–Tetrode [62] equation:

(16) $$\frac{S^{id}}{kN}=\ln\left(\frac{V}{N\;\Lambda^{3}}\right)+2.5$$

here k is the Boltzmann constant, N is the number of droplets, V is the total volume of the system, Λ is de Broglie wavelength and is expressed as follows:

(17) $$\Lambda =\frac{h}{\sqrt{2\pi \mu kT}}$$

where h is the Planck constant, μ is the mass of the oil droplet and T is temperature. In the literature on microemulsions, another equation the entropy of the system can be found [63], and it can be re-written in the following form:

(18) $$\frac{S^{mix}}{kN}=\ln\left(\frac{V}{N\;l^{3}}\right)+1$$

In equation (18), the parameter l is the length over which the droplet contour should be shifted to be counted as a new configuration [63]. Overbeek [64] also proposed an equation which can be re-written in a form that combines eqs (15), (18)), but the meaning of the length parameter would be close to the size of a water molecule. The difference in the last term of eqs (16), (18)) probably comes from counting the contribution from the translational motion in eq (16). Without an ambition to resolve the meaning of the *Λ* or *l* parameter, below we will show that the contribution from the entropy of oil droplets can cause partial emulsification of oil in water.

For the calculations, it is convenient to normalize the Gibbs energy (eq (14)) by volume rather than the number of particles. Technically, this can be done using the definition of the volume fraction $\varphi =\frac{v_{HS}N}{V}$. The enthalpic part of the free energy dependent on the surface tension and droplet size normalized per volume can be written as follows:

(19) $$\frac{\Delta H}{V}=\frac{\varphi }{v_{HS}}a\gamma_{ow}$$

In eq (19) a is the area of a hard sphere (droplet) and $v_{HS}$ is its volume. The volume-normalized Gibbs energy of the system for the hard sphere model is calculated in eq (20):

(20) $$\frac{\Delta G}{V}=\frac{\varphi }{v_{HS}}\left[a\gamma_{ow}-kT\left(\ln\frac{v_{HS}}{\varphi \Lambda^{3}}+\frac{5}{2}-\frac{4\varphi -3\varphi^{2}}{{\left(1-\varphi \right)}^{2}}\right)\right]$$

**Fig. 9:**
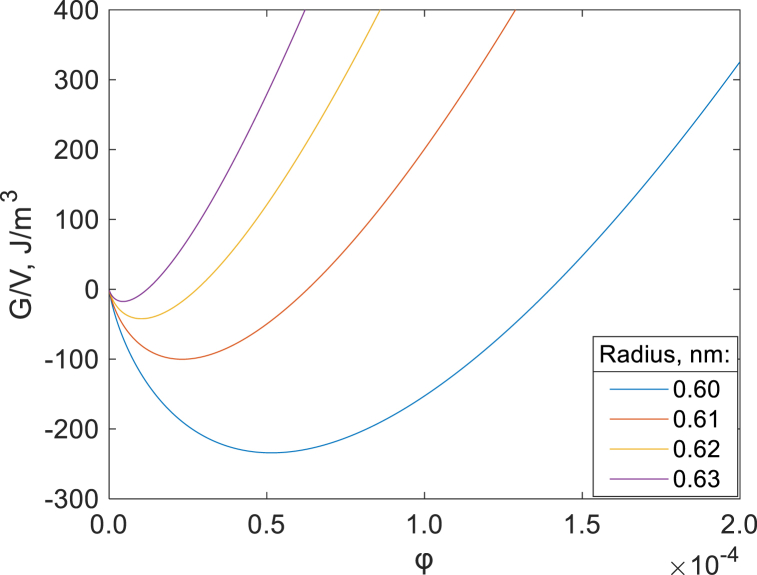
Gibbs free energy as a function of oil volume fraction (*φ*) in the system; results for different droplets sizes assuming a constant oil/water interfacial tension of 25 mJ/m^2^. Parameter Λ is calculated using eq 17.

Fig. 9 shows the calculated free energy of the system for droplets of different sizes for the hard sphere model (using de Broglie wavelength), while Fig. S21 shows the calculations for the microemulsion model with constant parameter l. For a system to be stable, the free energy of the system should be below zero. The system displayed here has an oil/water interfacial tension of 25 mJ/m^2^ [65]. A clear dependence on the droplet size and the volume fraction is shown. For the hard sphere (Fig. 9) case, droplets with radii above ≈0.63 nm cannot be thermodynamically stabilized at reasonable volume fractions. As the droplet size decreases, the free energy further decreases with stabilization. One should mention, however, that the Gibbs energy model used here does not take into account the effect of hydrophobic hydration, which predicts ordering of water molecules around hydrophobic cavities less than 1 nm in diameter [66]. Therefore, further decrease of Gibbs energy upon decrease of droplet size should not happen due to hydrophobic hydration effect.

Overall, regardless of the entropy expression and the value of Λ parameter, one should expect that certain amount of very small oil droplets can be thermodynamically stabilized in liquid water without addition of surfactant.

In this regime, TAP water has qualitatively the same behavior as the other water grades and quantitative results indicate that TAP water can be a more efficient solvent. To appreciate the special behavior of TAP, as evidenced by the high slope and apparent solubility values in Figs. S17 and 5b, its overall constitution compared to the other water grades should be considered. Apart from the electrolyte concentration, pH values are different [47]. Purified grades have a pH around 7 while the TAP water used in this work has a pH of 8.2 [47]. The slightly basic pH of TAP water can increase deprotonation of the free fatty acids of oil and make them act as surfactants and thus decrease $\gamma_{ow}$.

## 5 Conclusions

In this study we demonstrated a relation between different soil removal mechanisms when purified water grades are used. We suggest that the washing process consisting of a number of washing cycles can be divided into two regimes where different washing mechanisms prevail. The first, exponential, regime relies on a surface mechanism and results in high washing efficiency. Superior water purity further increases the efficiency of this regime. The second, linear, regime, is connected to bulk mechanisms and the total efficiency increases at a lower rate.

Overall, purified water grades outperformed TAP water in all temperatures measured. QCM-D results suggest that the main part of deposited Vaseline is removed during the first two washing cycles. Increased temperature promoted the removal of soil in all experiments.

We suggest that the volume of the oil left in the system after each cycle divides the washing process into two regimes where different washing mechanisms prevail. During the first regime, where the amount of oil is high, a roll up mechanism prevails and removes high amounts of oil. The absence of ions in the system will increase the electrostatic repulsion and will further facilitate the roll-up mechanism. As the oil volume decreases in the system, solubilization and formation of microemulsion droplets is of increasing importance and a new regime is established. This mechanism removes oil from the surface but at a lower rate.

Although the mechanisms used for washing and cleaning are known [1,27,30,32,36,37,67], a connection between them and the washing process with purified water grades was not investigated in detail. From our previous data [23], we demonstrated that pure water facilitates the roll-up mechanism by increasing the electrostatic repulsion between the surface and the soil. Here, we further expand our knowledge of the system, as we reveal that different mechanisms corresponding to different washing regimes are differently affected by water purity. Moreover, the knowledge of reversibility of the processes associated with the mechanisms will help to design optimal washing cycles.

Subsequent studies should be focused on implementing ultra-pure water in washing and cleaning different types of surfaces. The final goal of this study would be to understand the properties of washing with pure water in order to better utilize it in practical applications.

## Author contribution statement

Andriani Tsompou: Conceived and designed the experiments; Performed the experiments; Analyzed and interpreted the data; Wrote the paper.

Vitaly Kocherbitov: Conceived and designed the experiments; Analyzed and interpreted the data; Contributed reagents, materials, analysis tools or data; Wrote the paper.

## Data availability statement

Data included in article/supp. material/referenced in article.

## Declaration of competing interest

The authors declare that they have no known competing financial interests or personal relationships that could have appeared to influence the work reported in this paper
