# Supplementary material for: Surface and bulk mechanisms in repeating treatment of solid surfaces by purified water
Source: Heliyon. 2023 Jun 9;9(6):e17163. doi: 10.1016/j.heliyon.2023.e17163 (PMC10361314; doi:10.1016/j.heliyon.2023.e17163)
Supplement: Multimedia component 1 [file mmc1.pdf]

Supporting Material

Surface and bulk mechanisms in repeating treatment of solid surface by purified water

Andriani Tsompou[1,2] and Vitaly Kocherbitov[1,2*]

[1] Department of Biomedical Science, Malmö University, Malmö, Sweden

[2] Biofilms research center for Biointerfaces, Malmö University, Malmö, Sweden

*Corresponding author. Department of Biomedical Science, Malmö University, SE-20506, Malmö, Sweden. Phone: +4640-6657946. E-mail: Vitaly.Kocherbitov@mau.se

S1. Quartz Crystal Microbalance with Dissipation (QCM-D)

S.1.1 Raw data

Raw data for experiments with washing with MQ, DIRO, TAP, and NaCl at 25 and 40 °C for 4 washing cycles.

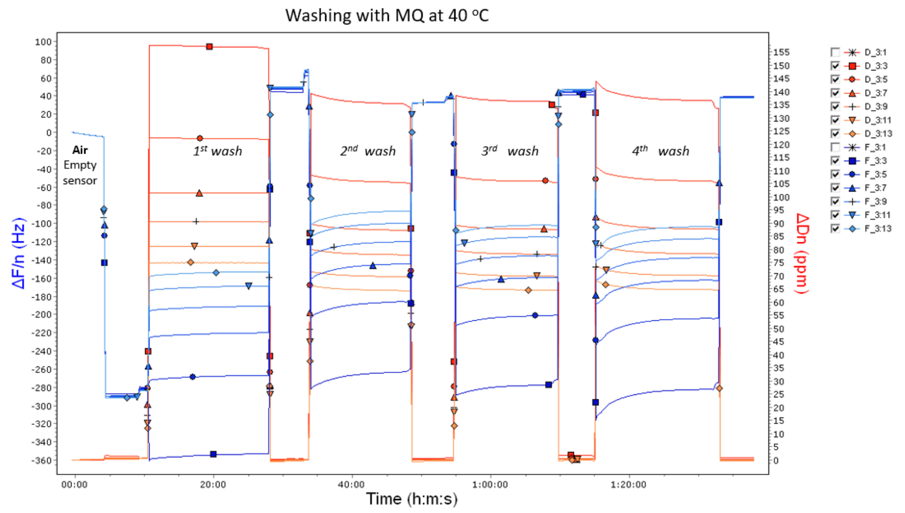

Figure S1: Frequency (blue) and dissipation (red) shifts for overtones 3,5,7,11,13 obtained from QCMD-D measurement of DIRO water at 25 °C. The regimes are: 1 – empty sensor in air, 2 – sensor coated with a vaseline thin film in air, 3 – coated sensor in DIRO water, 4 – dried sensor after treatment with water. Regimes 3 and 4 continue until the 4 washing cycles are completed.

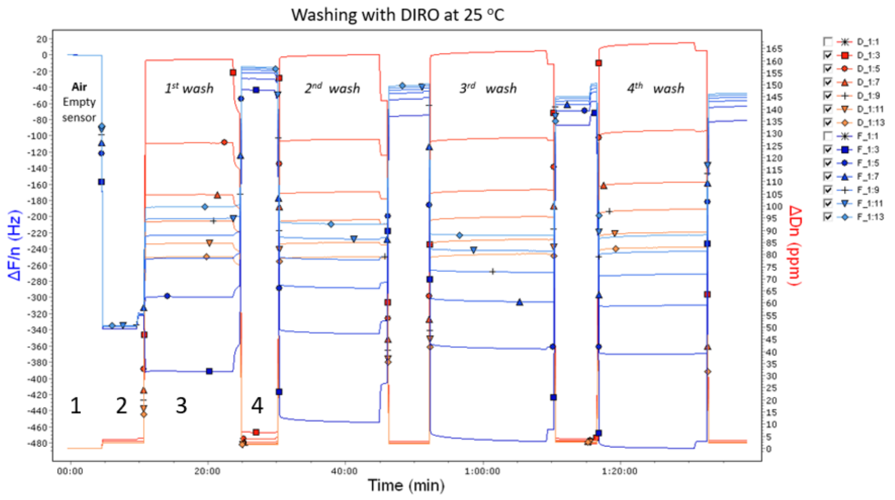

Figure S2: Frequency (blue) and dissipation (red) shifts for overtones 3,5,7,11,13 obtained from QCMD-D measurement of DIRO water at 25 °C. The regimes are: 1 – empty sensor in air, 2 – sensor coated with a vaseline thin film in air, 3 – coated sensor in DIRO water, 4 – dried sensor after treatment with water. Regimes 3 and 4 continue until the 4 washing cycles are completed.

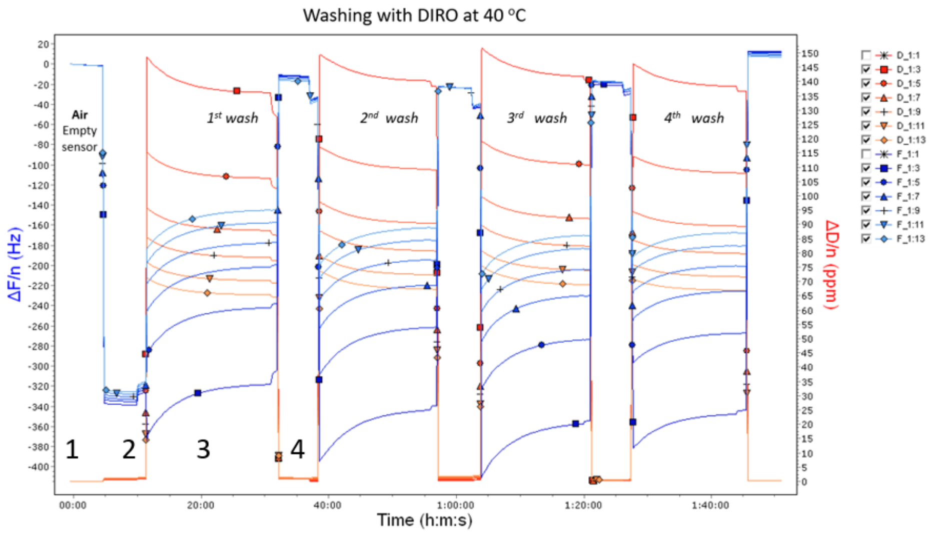

Figure S3: Frequency (blue) and dissipation (red) shifts for overtones 3,5,7,11,13 obtained from Type equation here.QCMD-D measurement of DIRO water at 40 °C. The regimes are: 1 – empty sensor in air, 2 – sensor coated with a vaseline thin film in air, 3 – coated sensor in DIRO water, 4 – dried sensor after treatment with water. Regimes 3 and 4 continue until the 4 washing cycles are completed.

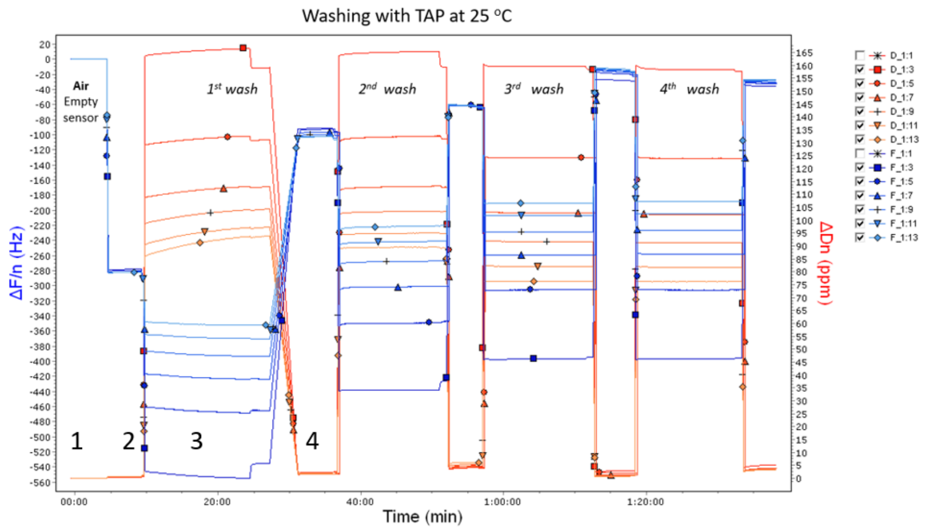

Figure S4: Frequency (blue) and dissipation (red) shifts for overtones 3,5,7,11,13 obtained from QCMD-D measurement of TAP water at 25 °C. The regimes are: 1 – empty sensor in air, 2 – sensor coated with a vaseline thin film in air, 3 – coated sensor in DIRO water, 4 – dried sensor after treatment with water. Regimes 3 and 4 continue until the 4 washing cycles are completed.

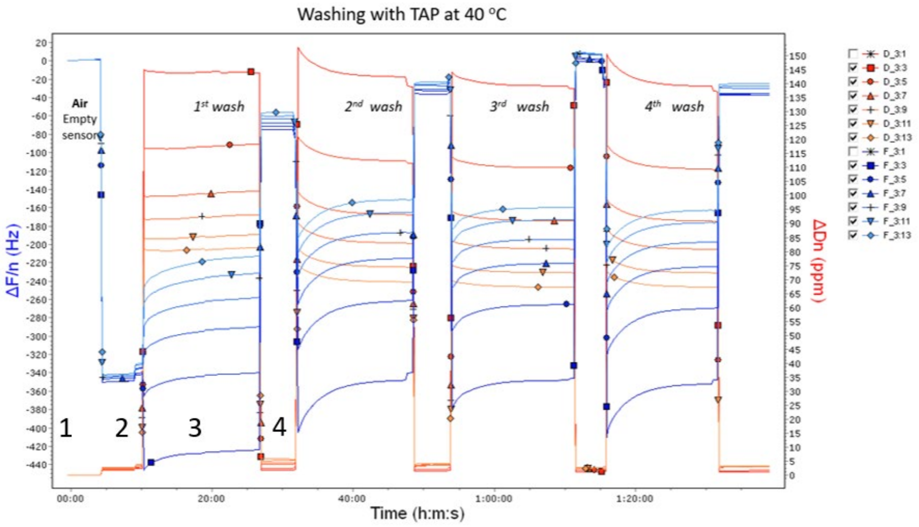

Figure S5: Frequency (blue) and dissipation (red) shifts for overtones 3,5,7,11,13 obtained from QCMD-D measurement of TAP water at 40 °C. The regimes are: 1 – empty sensor in air, 2 – sensor coated with a vaseline thin film in air, 3 – coated sensor in DIRO water, 4 – dried sensor after treatment with water. Regimes 3 and 4 continue until the 4 washing cycles are completed.

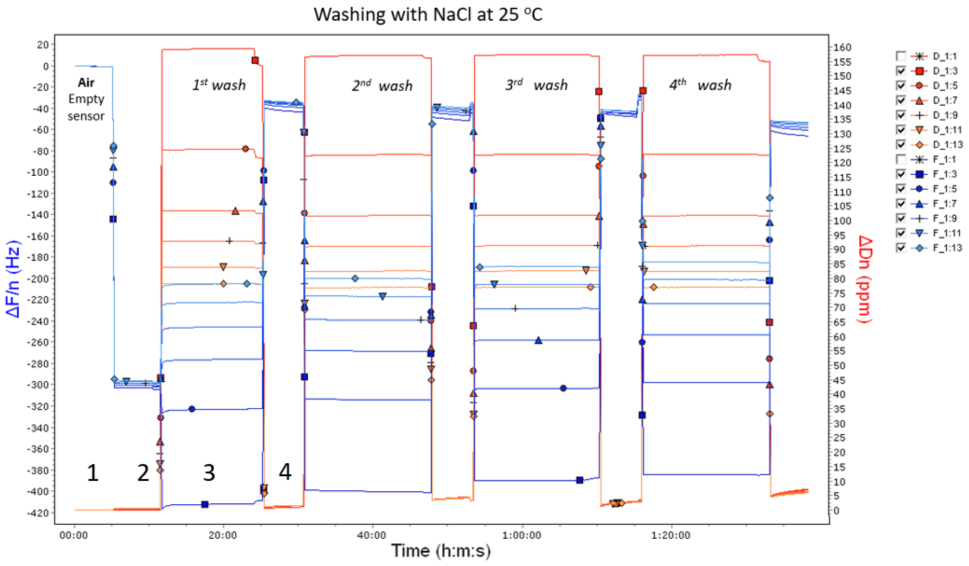

Figure S6: Frequency (blue) and dissipation (red) shifts for overtones 3,5,7,11,13 obtained from QCMD-D measurement of NaCl water at 25 °C. The regimes are: 1 – empty sensor in air, 2 – sensor coated with a vaseline thin film in air, 3 – coated sensor in DIRO water, 4 – dried sensor after treatment with water. Regimes 3 and 4 continue until the 4 washing cycles are completed.

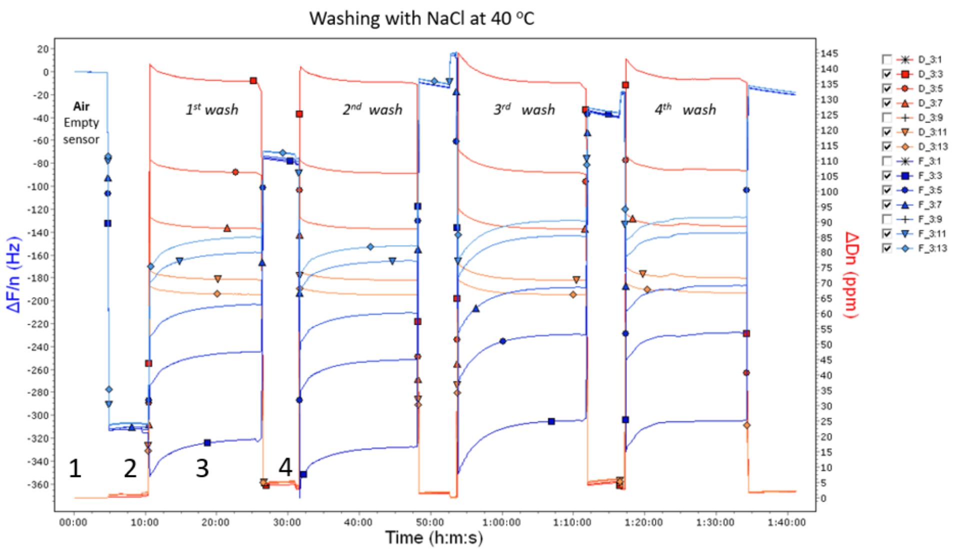

Figure S7: Frequency (blue) and dissipation (red) shifts for overtones 3,5,7,11,13 obtained from QCMD-D measurement of NaCl water at 40 °C. The regimes are: 1 – empty sensor in air, 2 – sensor coated with a vaseline thin film in air, 3 – coated sensor in DIRO water, 4 – dried sensor after treatment with water. Regimes 3 and 4 continue until the 4 washing cycles are completed.

S1.2 Characterization of vaseline film properties in air using QCM-D

The experimental output of QCM-D experiments are the frequency and dissipation shift that occur in all the overtones on the surface of the sensor. In all samples, the normalized frequency for each overtone, $\frac{\Delta f}{n}$, did not deviate strongly, thus the deposited layer can be considered as rigid and the Sauerbrey equation can be used to calculate the adsorbed mass and thickness[1].

For film deposition, the sensor should be removed from the machine and then mounted again. This process can create an additional 20 Hz error in frequency. To decrease the error, the bare and coated sensor were measured 5 times and the mean frequency values were calculated. The same process applied for both coated and treated sensor surfaces.

As mentioned in previous studies[2], the normalized frequency should exhibit a linear behavior to the squared overtone number. Figure S8 shows an example of this behavior for a coated sensor surface.

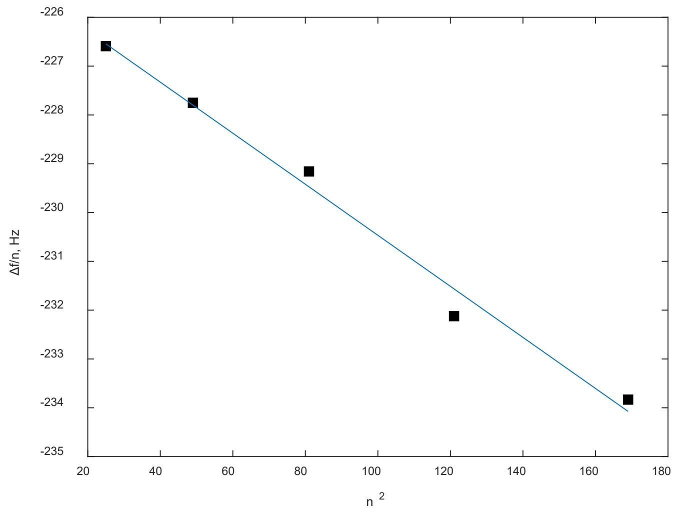

Figure S8: The normalized frequency as a function of squared overtone number for a thin film of Vaseline in air for an experiment with NaCl at 25 °C.

In particular, the dependence of the normalized frequency on the overtone number is expected to obey the following relation[3]:

$$\frac{\Delta f}{n} = \frac{-2f_0^2 m_f}{Z_q}\left(1 + \frac{1}{3}\frac{Z_q^2}{Z_f^2}\left(\frac{m_f}{m_q}n\pi\right)^2\right) \quad \text{(S1)}$$

where $Z_q = 8.8 * 10^6 kg\ m^{-2}s^{-1}$ – the acoustic or mechanical impedance of quartz; $Z_f = \sqrt{\rho(G' + G'')}$ – the acoustic impedance of the film; f_0 – the fundamental frequency; m_f – the mass of the film per unit area; m_q – is the areal mass density of the quartz crystal. Equation 4 can be presented in the following way:

$$\frac{\Delta f}{n} = -am_f\left(1 + bm_f^2 n^2\right) \quad \text{(S2)}$$

where $a = \frac{2f_0^2}{z_q}$ and $b = \frac{1}{3}\frac{z_q^2\pi^2}{z_f^2 m_q^2}$. Tables S1 and S2 demonstrate the calculated thickness of each film before and after washing using the Sauerbrey equation.

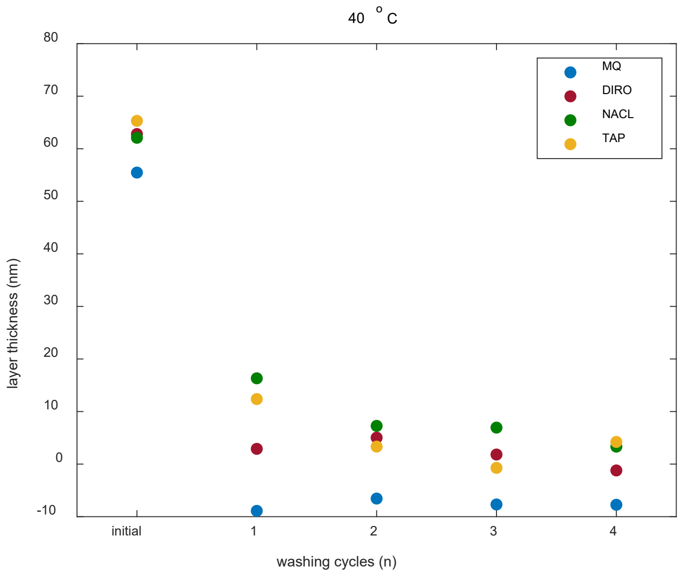

Figure S9: Film thickness after each washing cycle at 40 °C, calculated with the Sauerbrey equation

S1.3 Voigt model

Although most of the results exhibited a clear linear trend, indicative of a rigid film, there were some films with unclear properties. Either noticeable shifts in the dissipation (ΔDn) was observed, or the $\frac{\Delta f}{n}$ was different for each overtone. To fully understand the properties of the films and get more trustworthy data, the Voigt model, a viscoelastic model was also used. The expressions obtained by applying the Voigt model for a viscoelastic film adsorbed on a solid surface and immersed in a Newtonian fluid are:

$$\Delta f_n / f_n = - \frac{d_p \rho_p}{d_Q \rho_Q} \left[1 - \eta_\iota \rho_\iota \frac{\left(\eta_p / \rho_p \, \omega_n^2 \right)}{\mu_p^2 + \omega_p^2 \eta_p^2} \right] \quad \text{(S3)}$$

$$\Delta D_n = \frac{1}{d_Q \rho_Q} \left[\eta_\iota \rho_\iota \frac{d_p \mu_p \omega_n}{\mu_p^2 + \omega_p^2 \eta_p^2} \right] \quad \text{(S4)}$$

Where d, p and ω_n are the thickness, density and $2\pi f_n$ respectively. Q, p and l stand for quartz crystal, film and liquid medium, respectively. Overtones 3 to 13 were used to fit the data in the Voigt model through the QTools software. Tables X, X shows the thickness that was obtained alongside other viscoelastic properties of the films for both temperatures.

S1.3.1 Modeling the QCM-D data

Table S1: Fixed parameters used for modeling water grades in a coated senor. All water grades were modeled as a bulk fluid. Vaseline corresponds to layer 1. Overtones 3-13 were used for modeling.

Fixed parameters				
Water grades	Fluid density (kg/m^3)	Fluid thickness (m)	Fluid viscosity (mPa s)	Layer density (kg/m^3)
MQ	997	1	0.89	940
DIRO	997	1	0.89	940
TAP	997	1	0.89	940
10 mM NaCl	997	1	0.89	940

Table S2: Vaseline film thickness before, during and after water treatment at 25 °C calculated from QCM-D data using the Voigt model.

**Film thickness (nm)**	**Water grades**			
	MQ	DIRO	NaCl	TAP
**Coated film**	71.0	64.1	57.0	53.5
**1st washing cycle**	18.0	3.4	7.7	53.4
**Treated film (1)**	13.1	6.2	7.3	18.7
**2nd washing cycle**	4.5	11.6	8.8	12.0
**Treated film (2)**	$1.7\ e^{-14}$	11.3	8.8	12.1
**3rd washing cycle**	5.8	14.6	13.8	10.6
**Treated film (3)**	1.1	13.7	8.8	3.4
**4th washing cycle**	6.9	12.9	$4.7\ e^{-11}$	13.6
**Treated film (4)**	3.3	11.0	12.2	6.9

Table S3: Vaseline film thickness before, during and after water treatment at 40 °C calculated from QCM-D data using the Voigt model.

**Film thickness (nm)**	**Water grades**			
	MQ	DIRO	NaCl	TAP
**Coated film**	55.2	63.4	59.1	66.0
**1st washing cycle**				
**7.1**	$7.1\ e^{-4}$	2.6	14.7	13.7
**2nd washing cycle**				
**Treated film (2)**	$1.8\ e^{-14}$	4.6	2.2	6.1
**3rd washing cycle**				
**Treated film (3)**	$7.2\ e^{-4}$	3.6	7.4	$1.4\ e^{-3}$
**4th washing cycle**				
**Treated film (4)**	$7.2\ e^{-4}$	$1.8\ e^{-14}$	3.6	6.7

S1.4 Washing with different pump speed

To test if the speed of the pump is a factor that affects the washing efficiency, experiments were performed at 25 °C with two different pump speeds (0.25 ml/min and 0.5 ml/min). The pump speed regulated the water flow during the washing process. Figure S10 shows that when washing with 1 washing cycle, the efficiency is increased when DIRO and NaCl water were used. When MQ and TAP water were used, the efficiency does not indicate a significant increase. When the same experiment was conducted with MQ water with 3 washing cycles (Figure S11), there is no significant increased efficiency. Due to non-defining data, all further experiments were conducted with 0.25 ml/min.

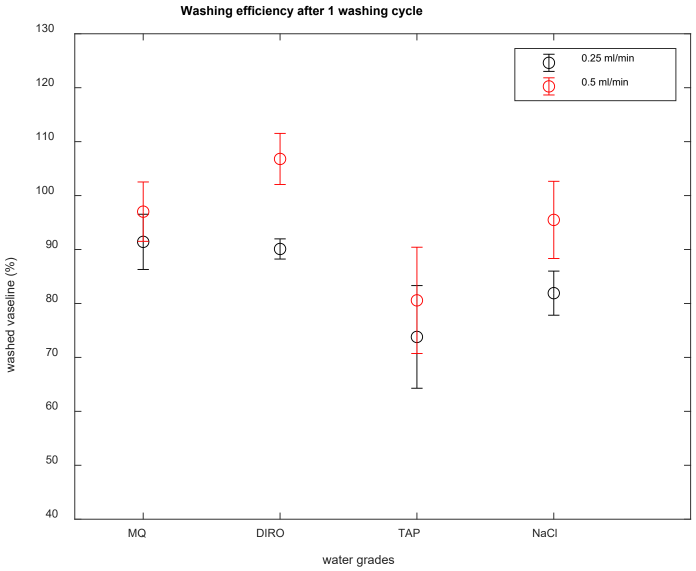

Figure S10: The cleaning efficiency: fraction of washed off Vaseline on QCM-D experiments when different water grades (MQ, DIRO, TAP and NaCl) were used t 25 °C at 0.25 ml/min (black) and 0.5 ml/min (red) pump speed to wash the surface with 1 washing cycle.

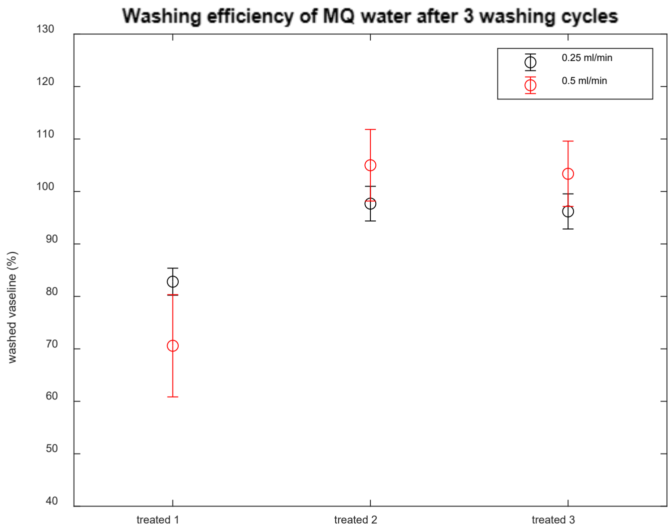

Figure S11: The cleaning efficiency: fraction of washed off Vaseline on QCM-D experiments when MQ was used t 25 °C at 0.25 ml/min (black) and 0.5 ml/min (red) pump speed to wash the surface with 3 washing cycles.

S1.5 Microscope images of silica surface after each washing cycle

The surface of the silica surface was monitored with an optical microscope after each step of the washing process. Once each washing cycles was completed, the surface was dried, and pictures were taken. After that, the new washing cycle started.

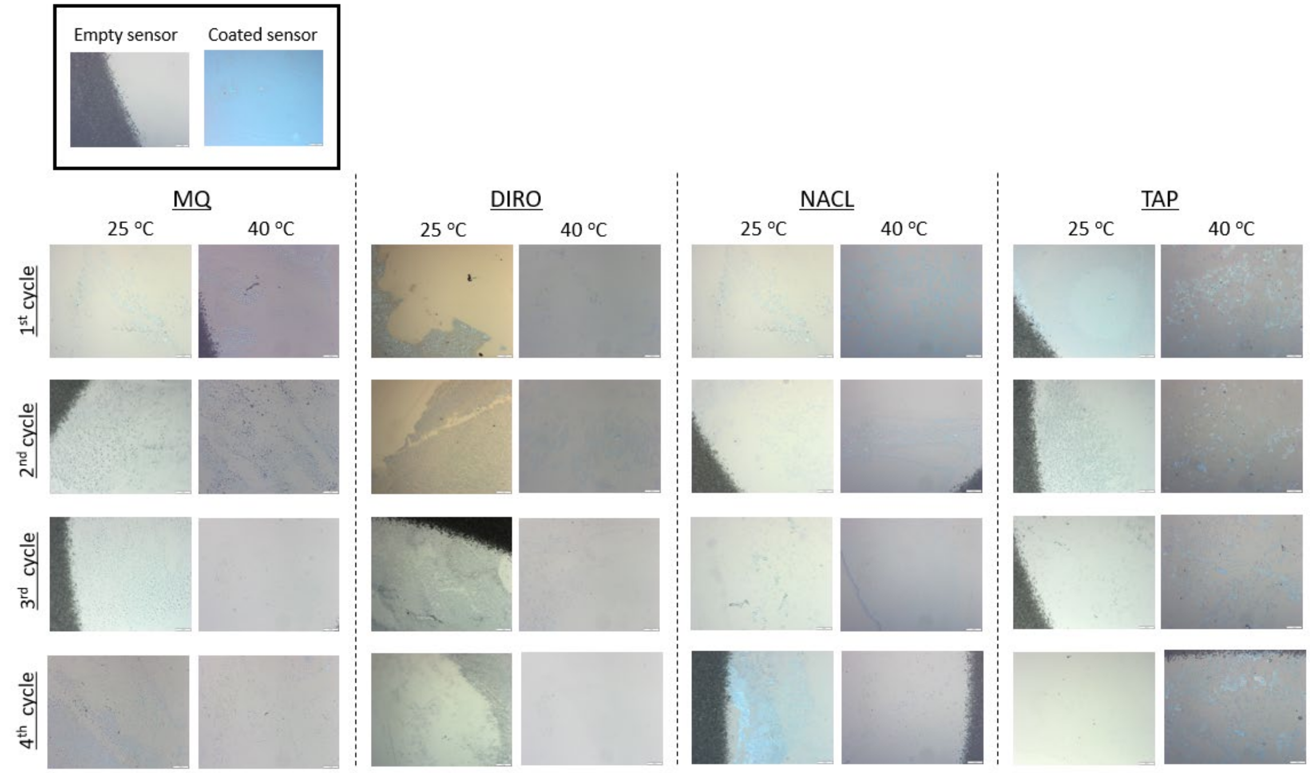

Figure S12: microscopy images of the sensor surface after each washing cycle. Scale bar: 100 µm. Images of the sensor surface before and after coating was also obtained (empty, coated sensor).

A) 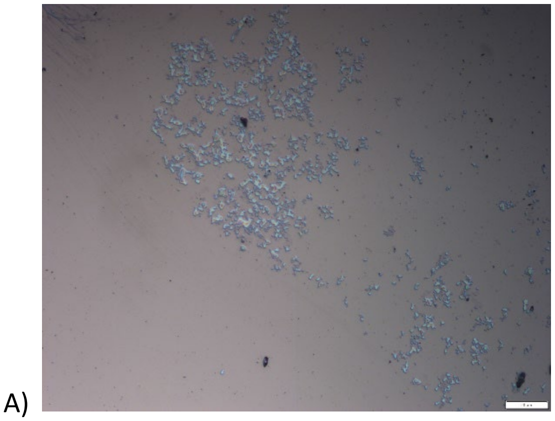

B) 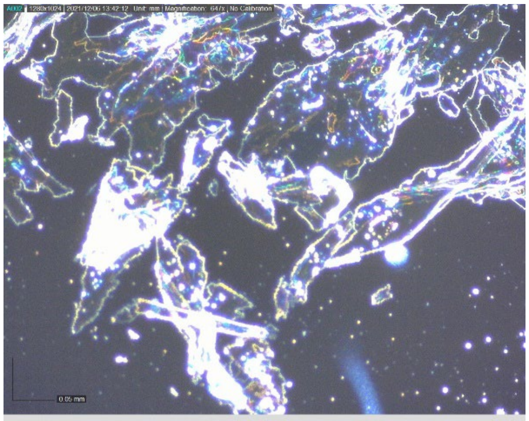

Figure S13: microscopy images of the sensor surface after washing with SDS at the cmc concentration (8 10^{-3} mol/L at 25 °C. A) Scale bar: 100 µm. B)Dino-lite digital microscope was used. Scale bar:50 µm

S2. Mass of oil before and after water contact (glass tubes)

S2.1 Effect of water purity and number of washing cycles

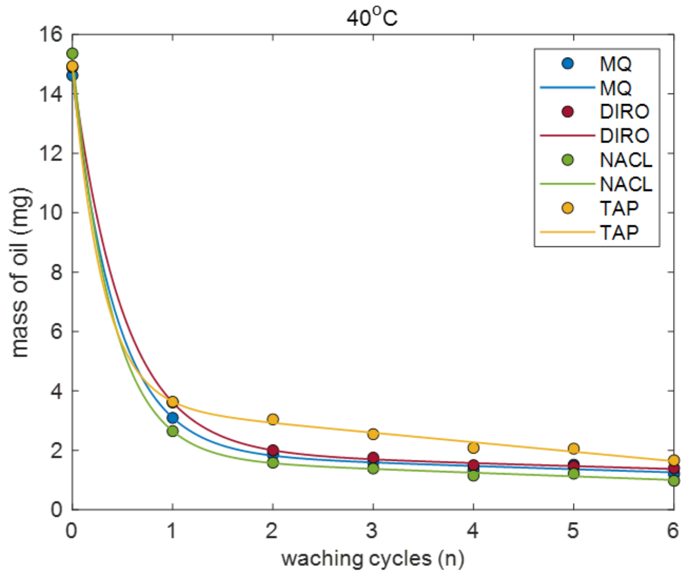

Figure S14: Average amount of olive oil left on the surface of glass tubes (mg) as a function of the number of washing cycles for MQ, DIRO, NaCl, and TAP at 40 °C. The number of replicates is 3. Plots with error bars can be found in the supplementary data.

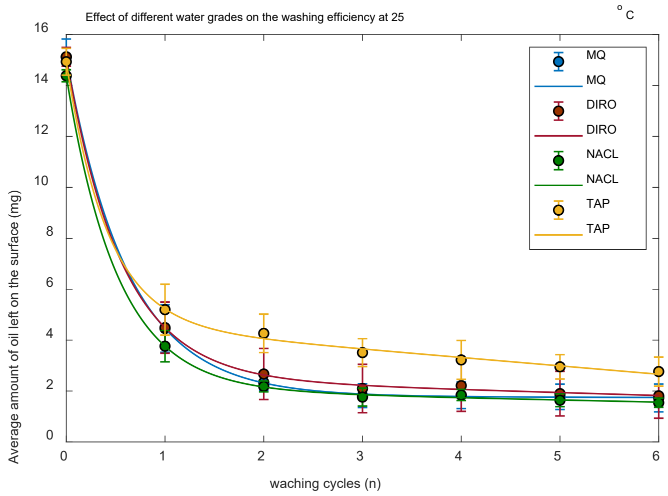

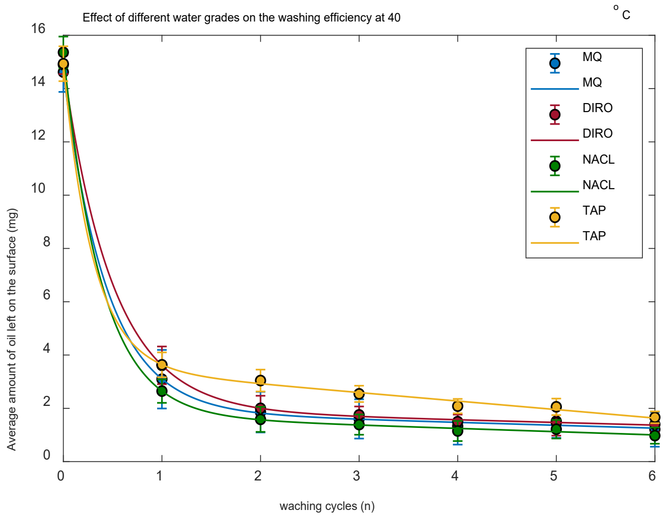

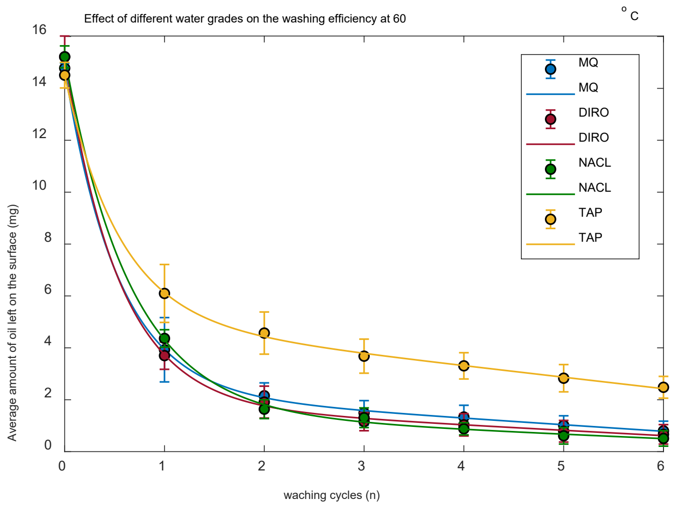

Figure S15: Average amount of oil left on the surface of glass tubes (mg) as a function of the number of washing cycles for MQ, DIRO, NaCl and TAP at (A) 25, (B) 40, and (C) 60 °C. The number of replicates is 3.

Table S4: Calculated values from non-linear fit of gravimetric data obtained from glass tubes for MQ, DIRO, NaCl and TAP at 25, 40, and 60 °C.

	m^o_s / m^o	c	a
M25	0.880772	1.608	-0.0007
M40	0.869758	2.2773	-0.0085
M60	0.844637	1.883	-0.0202
D25	0.833729	1.7954	-0.0091
D40	0.868298	1.9992	-0.0076
D60	0.878586	1.8536	-0.0154
N25	0.855715	1.9365	-0.0069
N40	0.887031	2.5762	-0.009
N60	0.901462	1.528	-0.0121
T25	0.689571	2.4113	-0.0319
T40	0.762248	3.3226	-0.028
T60	0.649774	1.8425	-0.0469

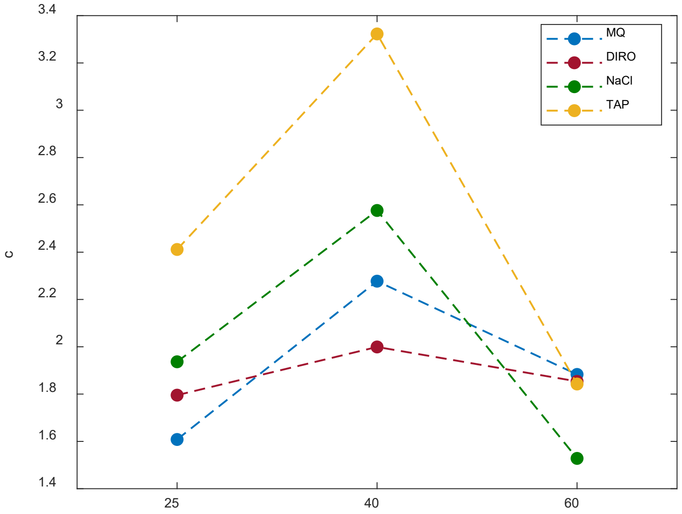

Figure S16: Calculated *c* is the exponential decay constant values from non-linear fit of gravimetric data obtained from glass tubes for MQ, DIRO, NaCl and TAP at 25, 40, and 60 °C

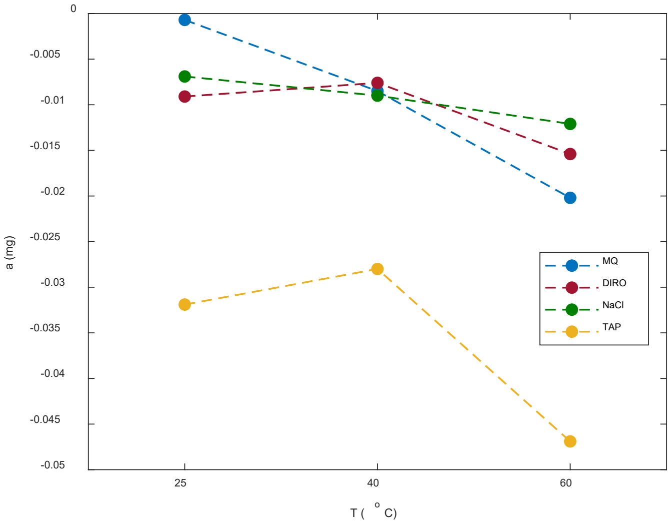

Figure S17: Calculated *a* (slope of the linear dependence) values from non-linear fit of gravimetric data obtained from glass tubes for MQ, DIRO, NaCl and TAP at 25, 40 and 60 °C

S2.2 Effect of temperature

A) B)

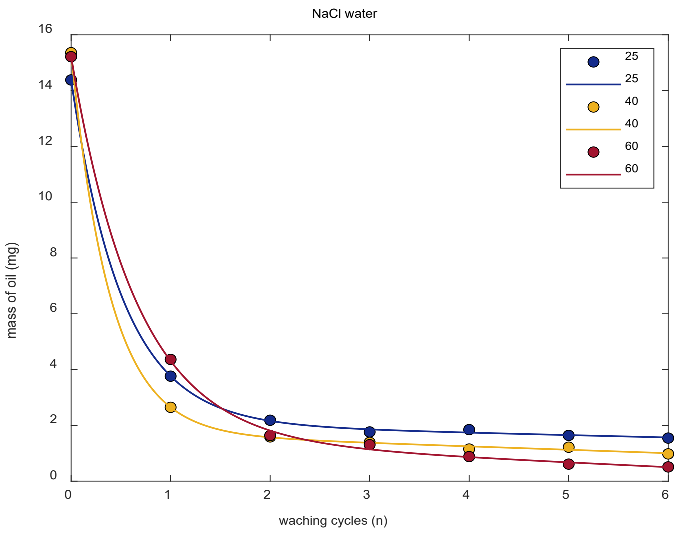

Figure S18: Average amount (mg) of oil left on the surface after each washing cycle when different water grades (a) NaCl (b) TAP were used at 25 °C , 40 °C, and at 60 °C. Data is non-linearly fitted using eq 4. The number of replicates is 3.

A) B)

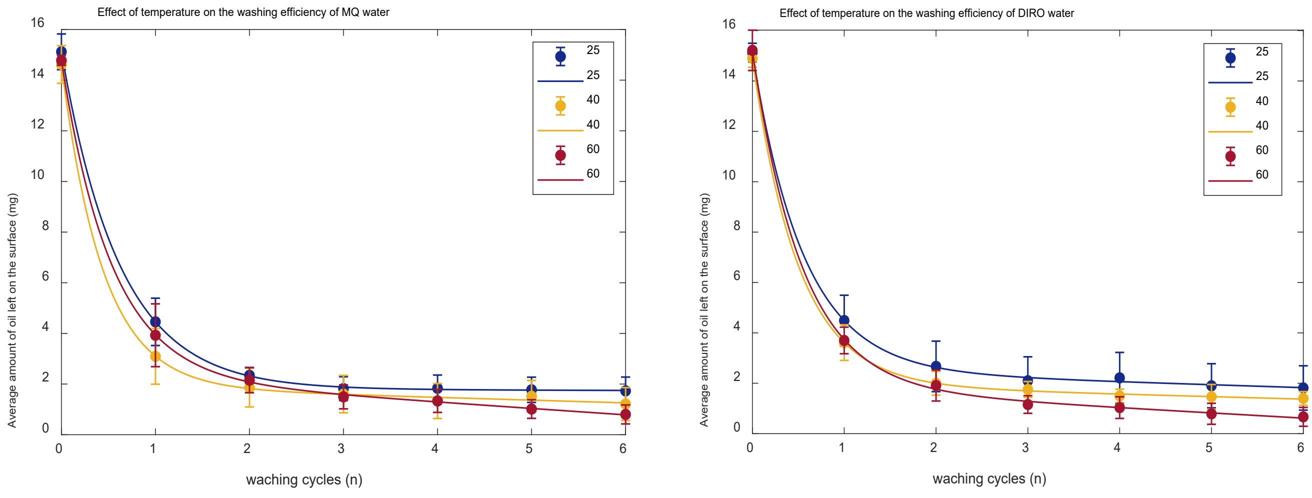

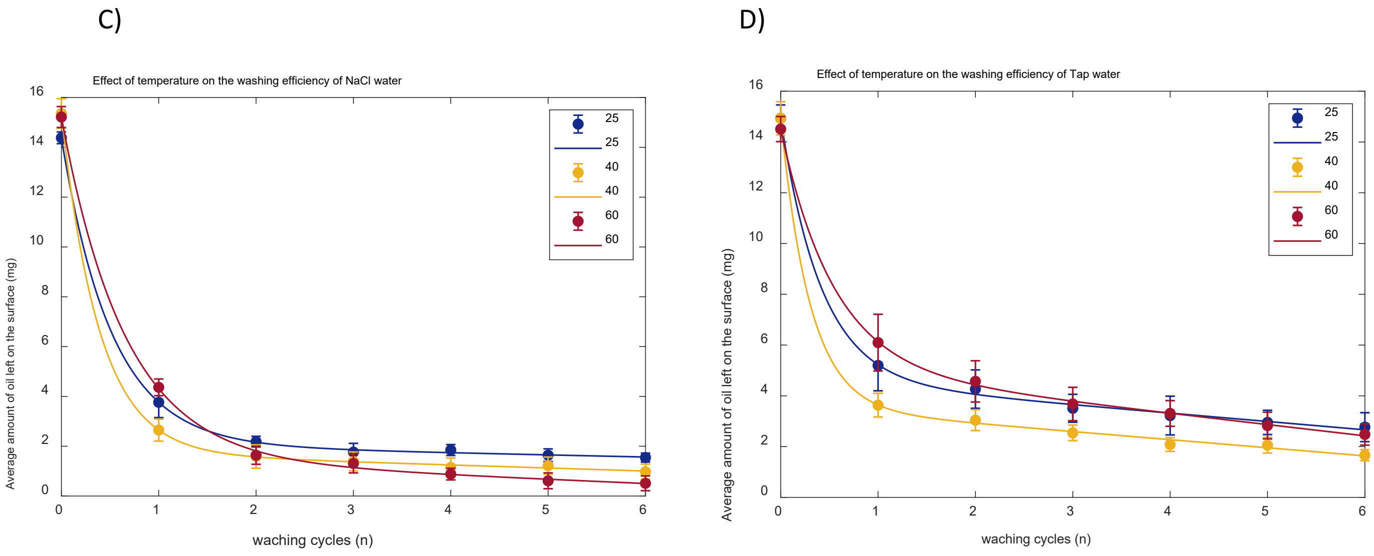

Figure S19: Average amount of oil left on the surface after each washing cycle when different water grades (A) MQ (B) DIRO (C) NaCl (D) TAP were used at 25 °C (blue), 40 °C (yellow), and at 60 °C (red). Data was non-linearly fitted as described in methods The number of replicates is 3.

S3: Surface and bulk mechanisms of washing

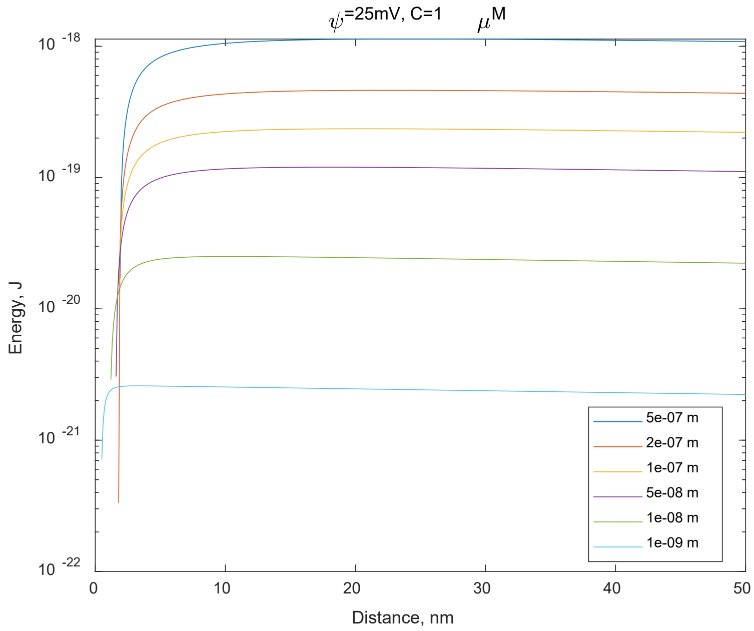

Figure S20. The two-particle DLVO energy as a function of distance for six different particle radii shown in the legend. The energy barrier decreases with decrease of the particle size and for the lowest curve (1 nm) the barrier is below the *kT* value for 298K (4.1×10^{-21} J)

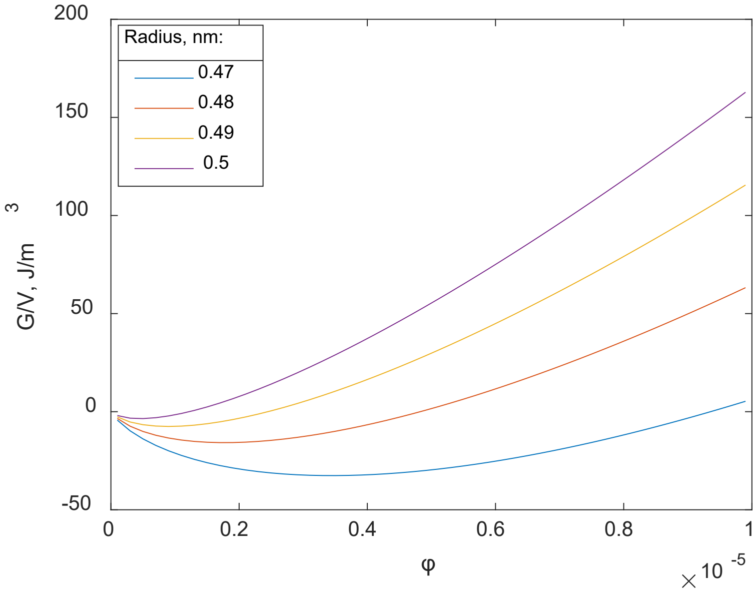

Figure S21. Gibbs free energy as a function of oil volume fraction (φ) in the system; results for different droplets sizes assuming a constant oil/water interfacial tension of 25 mJ/m^2. Parameter Λ is assumed to be equal to water molecule size of 3Å.

REFERENCES

1. Sauerbrey, G., *Verwendung von Schwingquarzen zur Wägung dünner Schichten und zur Mikrowägung.* Zeitschrift für physik, 1959. **155**(2): p. 206-222.
2. Tsompou, A. and V. Kocherbitov, *The effects of water purity on removal of hydrophobic substances from solid surfaces without surfactants.* Journal of Colloid and Interface Science, 2022. **608**: p. 1929-1941.
3. Znamenskaya, Y., et al., *Effect of hydration on structural and thermodynamic properties of pig gastric and bovine submaxillary gland mucins.* The Journal of Physical Chemistry B, 2012. **116**(16): p. 5047-5055.
